# Physalins V-IX, 16,24-*cyclo*-13,14-*seco* withanolides from *Physalis angulata* and their antiproliferative and anti-inflammatory activities

**DOI:** 10.1038/s41598-017-03849-9

**Published:** 2017-06-22

**Authors:** Cheng-Peng Sun, Chong-Yue Qiu, Feng Zhao, Ning Kang, Li-Xia Chen, Feng Qiu

**Affiliations:** 1 Tianjin State Key Laboratory of Modern Chinese Medicine and School of Chinese Materia Medica, Tianjin University of Traditional Chinese Medicine, 312 Anshanxi Road, Nankai District, Tianjin 300193 China; 2 Department of Natural Products Chemistry, School of Traditional Chinese Materia Medica, Key Laboratory of Structure-Based Drug Design & Discovery, Ministry of Education, Shenyang Pharmaceutical University, Shenyang, 110016 China; 3 School of Pharmacy, Key Laboratory of Molecular Pharmacology and Drug Evaluation (Yantai University), Ministry of Education, Collaborative Innovation Center of Advanced Drug Delivery System and Biotech Drugs in Universities of Shandong, Yantai University, Yantai, 264005 China

## Abstract

Five new physalins, including a novel 1,10-*seco* one, physalin V (**1**), a tricarboxylic acid cycle one, physalin VIII (**5**), a rare 11,15-*cyclo* one, physalin IX (**6**), and two new ones, physalins VI (**2**) and VII (**4**) were isolated from stems and leaves of *Physalis angulata* together with eleven known analogues (**3** and **7–16**). Their structures were established by MS, IR, UV, and NMR spectroscopic analysis, together with the X-ray diffraction analysis of neophysalin, physalin P (**12**), and the structure of physalin D_1_ (**3**) has been revised here. These isolated compounds were evaluated for their antiproliferative activities against human cancer cells (C4-2B, 22Rv1, 786-O, A-498, ACHN, and A375-S2) and inhibitory effects on nitric oxide production. Compounds **9** and **10** showed antiproliferative activities against all tested human cancer cells with IC_50_ values of 0.24–3.17 *μ*M. Compounds **1**, **3**, **4**, **9**, **10**, **13**, **14**, and **16** exhibited inhibitory activities against NO production. The IC_50_ values of compounds **9**, **10**, **13**, and **16** were between 0.32 and 4.03 *μ*M, while compounds **1**, **3**, **4**, and **14** had IC_50_ values of 12.83–34.19 *μ*M. Herein, plausible biosynthetic pathways for rare structures **1** and **6** and structure−activity relationships on the inhibition of NO production for all isolated compounds are discussed.

## Introduction

The withanolides are a group of natural C_28_ steroids with a *γ*- or *δ*-lactone based on an ergostane skeleton, which are derived from a parent 23-hydroxy-26-oic or 22-hydroxy-26-oic acid. They can be further divided into 22 subtypes based on the difference of the structural skeleton, such as normal withanolides, physalins, withaphysalins, neophysalins, jaborols, and so on^1, 2^. Physalins, commonly termed 16,24-*cyclo*-13,14-*seco* steroids, are classified as a group of withanolides with the most advanced oxidation level, from which they are formally derived from oxidative bond cleavage between C-13 and C-14 to produce a nine-membered ring, formation of a new six-membered carbocycle between C-16 and C-24, oxidation of the C-18 methyl group to a COOH group, which leads to 18,20-lactonization, and formation of an oxo bridge between C-14 and C-17, resulting in an oxygen heterocyclic system across rings C and D^3–5^.

**Figure 1:**
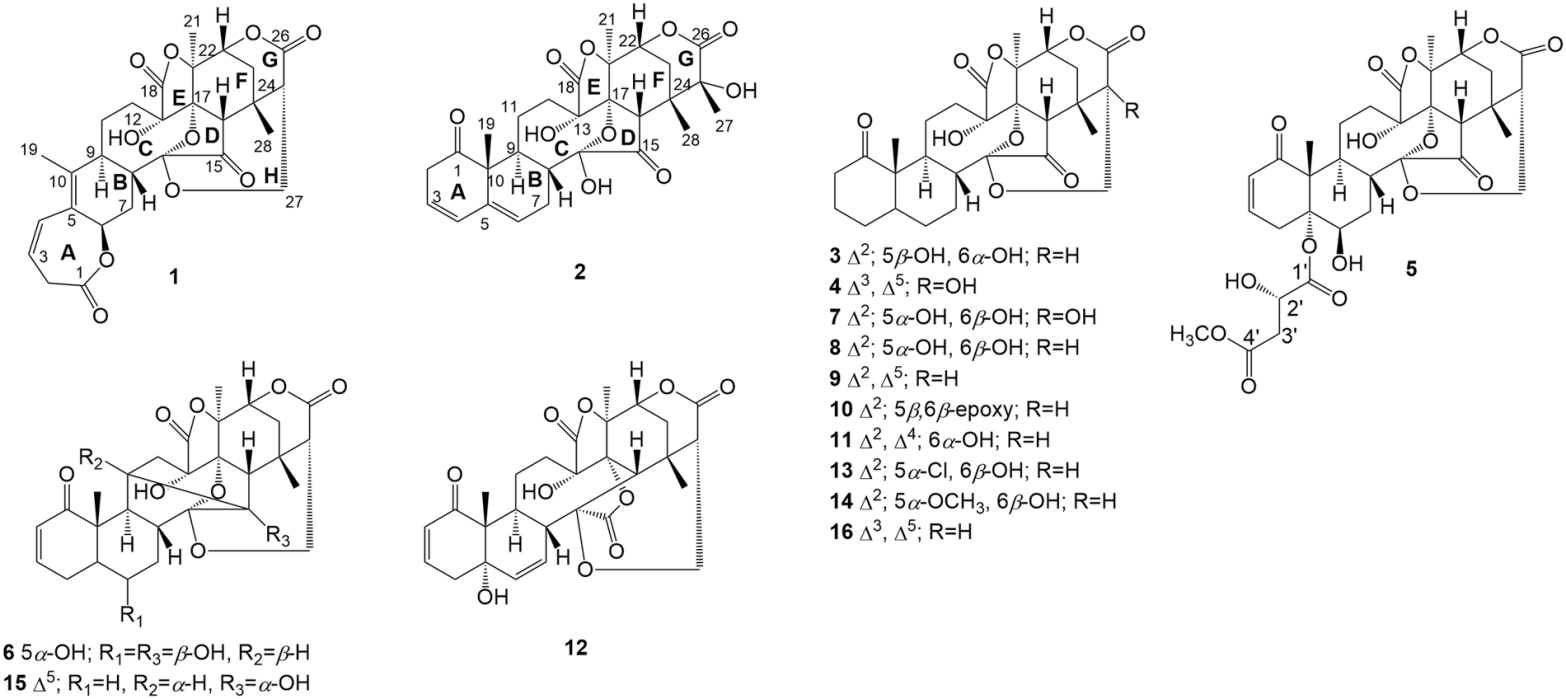
Chemical constituents of *P*. *angulata*.

The genus *Physalis* has attracted attention from scientists due to the occurrence of the first 16,24-*cyclo*-13,14-*seco* withanolide physalin A from *Physalis alkekengi* var. *franchetii* in 1969^6^. Over the past 47 years, about 60 physalins have been isolated from this genus. The genus *Physalis* (Solanaceae) comprising approximately 120 species, is widely distributed in subtropical and tropical regions all over the world. *Physalis angulata* L., known as *ku-zhi* in China^7^, is a folk medicine that has been used to treat a variety of illnesses in many countries, such as dermatitis, trachitis, impaludism, rheumatism, and hepatitis. It is also used as a diuretic, antipyretic, antileukemic, anticancer, and immuno-modulatory agents^8^. Phytochemical investigations of *P*. *angulata* have led to the isolation of many physalins and normal withanolides, such as physalins A, B, D, E, F, G, I, and H, and physagulins A, B, C, and F, and some of them displayed remarkable anti-inflammatory^9–12^, antitumor^13–16^, antinociceptive^17^, and immunomodulatory^18^ activities. As part of our ongoing research on isolating bioactive physalins from the genus *Physalis* to provide potential anticancer and anti-inflammatory medicines^11, 12, 15, 16^, the EtOH extracts of the dried stems and leaves of *P*. *angulata* were isolated to afford a novel 1,10-*seco* physalin, physalin V (**1**), a tricarboxylic acid cycle one, physalin VIII (**5**), a rare 11,15-*cyclo* one, physalin IX (**6**), and two new ones, physalins VI (**2**) and VII (**4**), together with eleven known analogues (Fig. 1), and the structure of the known physalin D_1_ (**3**) was revised. In this paper, we describe the isolation and structural elucidation of these compounds together with their antiproliferative and anti-inflammatory evaluations *in vitro*. Furthermore, biosynthetic pathways for the rare physalins **1** and **6** are proposed, and structure–activity relationships for all isolated compounds are preliminarily discussed.

## Results and Discussion

The EtOH extracts of stems and leaves of *P*. *angulata* were separated by silica gel, Sephadex LH-20, ODS open column chromatography, preparative TLC, and preparative HPLC to yield five new physalins, including an uncommon 1,10-*seco* one, physalin V (**1**), physalins VI (**2**), VII (**4**), and VIII (**5**), and a novel 11,15-*cyclo* one, physalin IX (**6**), together with eleven known analogues, 25*β*-hydroxyphysalin D (**7**)^19^, physalins D_1_ (**3**), D (**8**)^20^, B (**9**)^21^, F (**10**)^22^, G (**11**)^23^, P (**12**)^24^, H (**13**)^25^, I (**14**)^20^, and R (**15**)^26^, and isophysalin B (**16**)^25^ (Fig. 1).

### Structure elucidation

**Figure 2:**
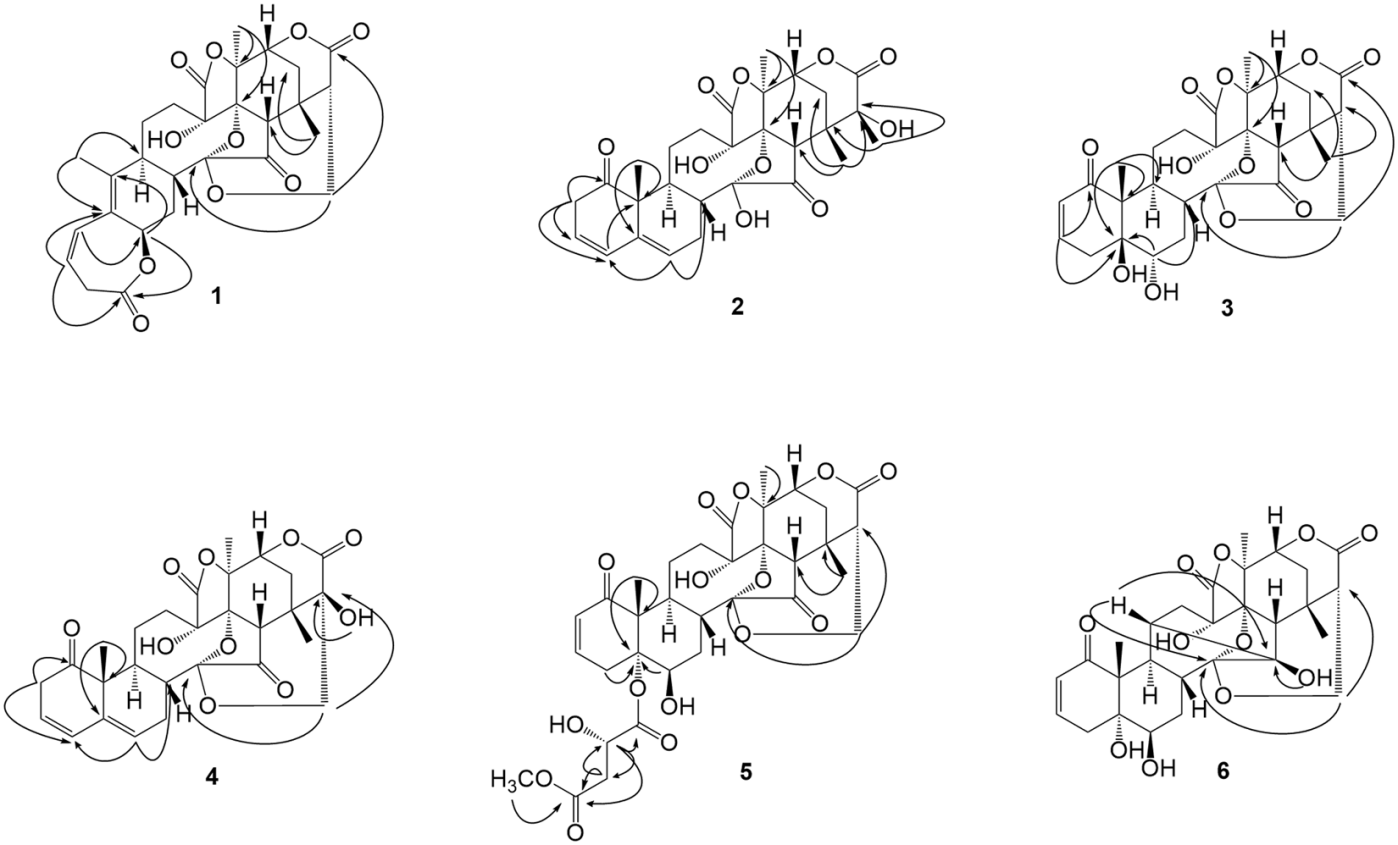
Selected HMBC correlations of compounds **1–6**.

**Figure 3:**
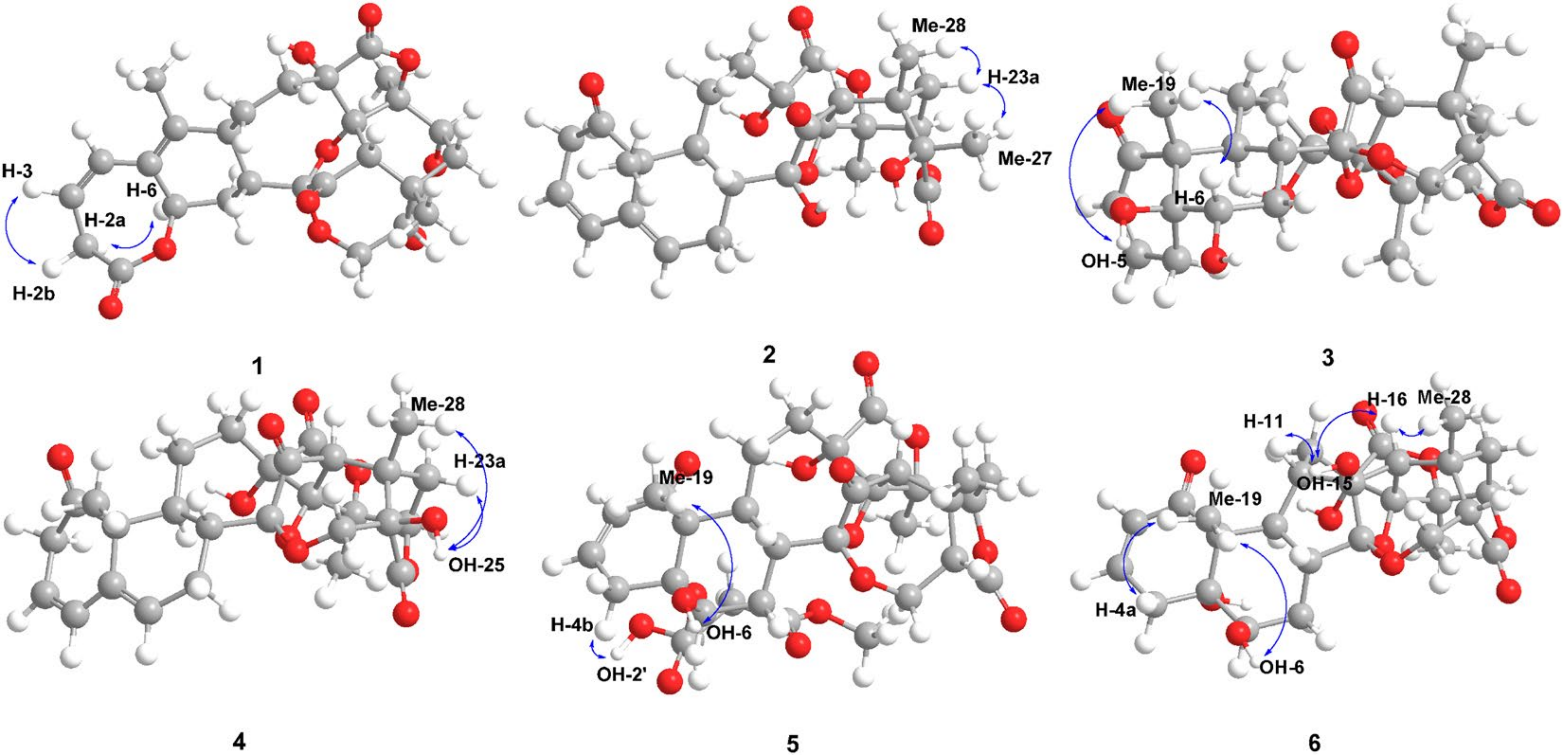
Selected NOESY correlations of compounds **1–6**.

**Figure 4:**
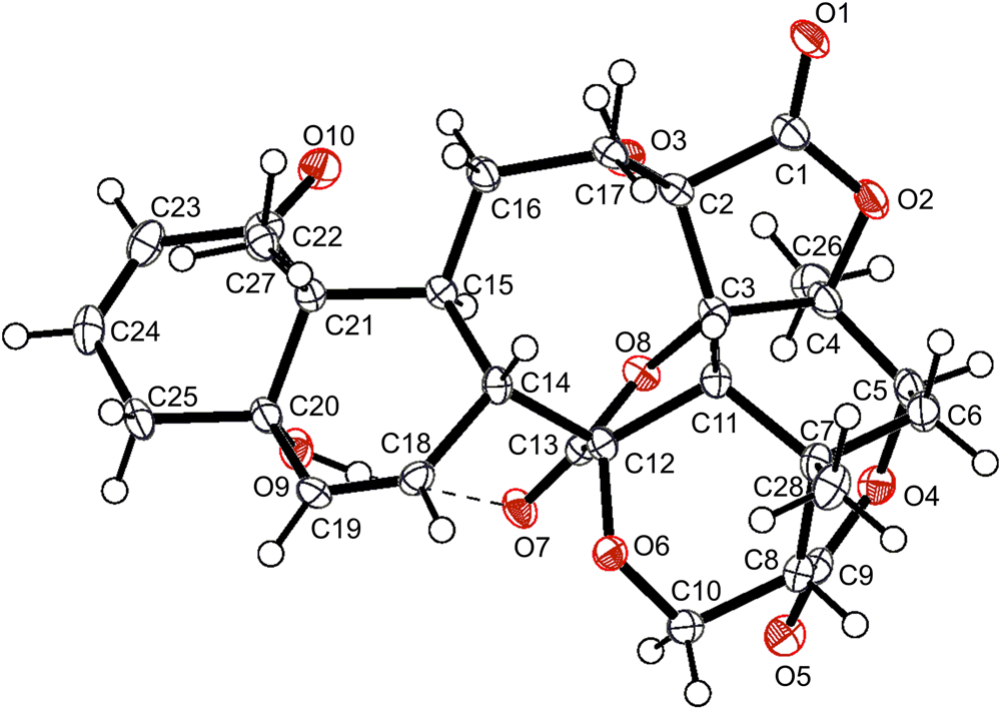
ORTEP drawing of compound **12**.

Physalin V (**1**) was isolated as an amorphous powder, and HRESIMS spectrum (Supplementary Fig. 8) showed that the quasi-molecular peak at *m*/*z* 549.1750 [M + Na]^+^ (calcd for C_28_H_30_O_10_Na, 549.1737), requiring the molecular formula C_28_H_30_O_10_. The IR spectrum (Supplementary Fig. 9) of **1** showed the presence of hydroxy (3400 cm^−1^), carbonyl (1728 cm^−1^), and olefinic (1646 cm^−1^) functionalities. The ^1^H NMR spectrum (Supplementary Fig. 1) of **1** displayed signals for three methyl groups at *δ*_H_ 1.83 (3 H, s, Me-19), 1.82 (3 H, s, Me-21), and 1.17 (3 H, s, Me-28), and two olefinic protons at *δ*_H_ 6.49 (1 H, dd, *J* = 11.2, 2.0 Hz, H-4) and 5.49 (1 H, td, *J* = 9.3, 2.0 Hz, H-3). The ^13^C NMR data (Supplementary Fig. 2) showed 28 carbon resonances, including one carbonyl carbon (*δ*_C_ 209.0), three hydroxycarbonyl carbons (*δ*_C_ 171.80, 171.79, and 167.2), four olefinic carbons (*δ*_C_ 141.0, 128.8, 125.7, and 118.3), one ketal carbon (*δ*_C_ 105.2), six oxygenated carbons (*δ*_C_ 80.5, 80.3, 78.8, 76.4, 71.2, and 61.2), and three methyl carbons (*δ*_C_ 24.3, 21.3, and 17.1). The ^1^H and ^13^C NMR data of **1** were closely resembled those of physalin G (**11**) isolated from *P*. *angulata* ^23^, with the exception that signals of ring A [*δ*_H_ 6.49 (1 H, dd, *J* = 11.2, 2.0 Hz, H-4), 5.49 (1 H, ddd, *J* = 11.2, 9.3, 2.0 Hz, H-3), and 5.25 (1 H, br s, H-6); *δ*_C_ 171.8 (C-1), 141.0 (C-10), 128.8 (C-4), 125.7 (C-5), 118.3 (C-3), 71.2 (C-6), and 35.6 (C-2)] were present in **1**, indicating that **1** was a 1,10-*seco* physalin^27, 28^. This conclusion was confirmed by the HMBC correlations from H-2a to C-1/C-3/C-4, H-3 to C-1/C-2/C-5, H-4 to C-2/C-5/C-6/C-10, and H-6 to C-1/C-5/C-8/C-10 (Supplementary Fig. 3–5), suggesting the presence of the seven-membered *β*,*γ*-unsaturated lactonic ring (Fig. 2). The relative configuration of **1** was established through the NOESY correlation of H-6 with H-2a (Fig. 3, Supplementary Fig. 6 and 7) in combination with the coupling constant (*J*_2a,3_ = 4.7 Hz), requiring *a*-orientations of H-6 and H-2a^27, 28^. According to X-ray diffraction data (Cu Ka) of physalin P (**12**) (Fig. 4), an acid induced benzilic acid-type rearranged product of physalins^29^, in combination with the biogenetic grounds and previous literatures^30–34^, **1** was assigned as (6*R*,8*R*,9*S*,13*S*,14*R*,16*S*,17*R*,20*S*,22*R*,24*S*,25*S*)-1,10-*seco* physalin G.

Physalin VI (**2**) was obtained as an amorphous powder with the molecular formula C_28_H_32_O_10_ based on HRESIMS *m/z* 527.1918 [M − H]^−^ (calcd for C_28_H_31_O_10_, 527.1917; Supplementary Fig. 17) and ^13^C NMR data (Supplementary Fig. 11). The IR spectrum (Supplementary Fig. 18) displayed absorption bands corresponding to hydroxy (3397 cm^−1^), carbonyl (1716 cm^−1^), and olefinic (1646 cm^−1^) functionalities. The ^1^H NMR data (Supplementary Fig. 10) of **2** observed three olefinic protons at *δ*_H_ 6.07 (1 H, br d, *J* = 11.2 Hz, H-4), 5.70 (1 H, br d, *J* = 4.8 Hz, H-6), and 5.67 (1 H, m, H-3), four methyl groups at *δ*_H_ 1.75 (3 H, s, Me-21), 1.43 (3 H, s, Me-28), 1.36 (3 H, s, Me-27), and 1.17 (3 H, s, Me-19), and the reduced two geminal oxymethylene protons in conjunction with the ^13^C NMR data [*δ*_C_ 209.6 (C-1), 140.4 (C-5), 128.0 (C-4), 126.6 (C-6), 122.6 (C-3), 101.0 (C-14), 22.4 (C-28), 20.7 (C-21), 19.4 (C-27), and 17.8 (C-19)], indicating the existence of a 1-oxo-3,5-diene unit and the nonexistence of a C(14)-O-C(27) cyclization moiety. The above NMR spectroscopic data of **2** were related to those of physalin M isolated from *P*. *alkekengi* L. var. *franchetii* ^35^, except for the presence of a methyl group [*δ*_H_ 1.36 (3 H, s, Me-27); *δ*_C_ 19.4 (C-27)] and an oxygenated carbon [*δ*_C_ 72.6 (C-25)], with the assumption for the presence of a hydroxy group at C-25. The key HMBC correlations from Me-27 to C-24/C-25/C-26 and Me-28 to C-16/C-23/C-24/C-25 (Fig. 2 and Supplementary Fig. 12–14) confirmed the hypothesis. The orientation of OH-25 was deduced to be *α* by NOESY correlations of Me-27 with H-23a and Me-28 with H-23a (Fig. 3 and Supplementary Fig. 15 and 16). Therefore, **2** was determined as (8*R*,9*S*,10*R*,13*S*,14*R*,16*S*,17*R*,20*S*,22*R*,24*S*,25*R*)-25-hydroxyphysalin M.

Physalin D_1_ (**3**) had the molecular formula C_28_H_32_O_11_ identified by HRESIMS *m/z* 543.1870 [M − H]^−^ (calcd for C_28_H_31_O_11_, 543.1866; Supplementary Fig. 26). Compound **3** exhibited the ^1^H and ^13^C NMR data (Supplementary Fig. 19–23) completely identical to those of physalin D_1_ isolated from *P*. *alkekengi* L. var. *franchetii* ^36^, indicating that they were the same compound. However, the NOESY spectrum of **3** displayed a key correlation between OH-5 and Me-19 (Fig. 3 and Supplementary Fig. 24 and 25), indicating a *β*-orientation of OH-5. This conclusion was further confirmed by the ^13^C NMR chemical shift value of the methyl group at C-10 [*δ*_C_ 8.5 (C-19)], since this value in 5,6-dihydroxy or 4,5,6-trihydroxy withanolides could indicate the relationships between rings A and B to be *cis*- (around *δ*_C_ 10) or *trans*-fusion (around *δ*_C_ 15)^37^. Hence, its structure was revised as (5*S*,6*S*,8*R*,9*S*,10*R*,13*S*,14*R*,16*S*,17*R*,20*S*,22*R*,24*S*,25*S*)-5,6-dihydroxyphysalin D.

The molecular formula of physalin VII (**4**) was determined as C_28_H_30_O_10_ based on HRESIMS *m/z* 525.1769 [M − H]^−^ (calcd for C_28_H_29_O_10_, 525.1761; Supplementary Fig. 35) and ^13^C NMR data (Supplementary Fig. 29). Comparison of the NMR data of **4** and isophysalin B (**16**)^25^ indicated that the proton at C-25 was replaced by a hydroxy group, since the chemical shift value of C-25 was deshielded from *δ*_C_ 50.9 in **16** to *δ*_C_ 73.6 in **4**, which was further supported by the HMBC correlations from OH-25 to C-25, H-27a/H-27b to C-14/C-24/C-25/C-26, and Me-28 to C-16/C-23/C-24/C-25 (Fig. 2 and Supplementary Fig. 30–32). Furthermore, the NOESY correlations of OH-25 with H-23a/Me-28 (Fig. 3 and Supplementary Fig. 33 and 34) suggested that OH-25 has the same orientation as Me-28 with *β*. Thus, **4** was established as (8*R*,9*S*,10*R*,13*S*,14*R*,16*S*,17*R*,20*S*,22*R*,24*S*,25*R*)-25-hydroxyisophysalin B.

HRESIMS analysis of physalin VIII (**5**) established the molecular formula C_33_H_38_O_15_ [m/z 673.2127 [M−H]^−^ (calcd for C_33_H_37_O_15_, 673.2132)] (Supplementary Fig. 45), indicating extra five carbons except for the C_28_ skeleton of the physalins. The detailed analysis of ^1^H and ^13^C NMR data (Supplementary Fig. 37 and 38) for **5** and physalin D (**8**)^20^ revealed that **5** possessed a similar structure to **8**, and the only difference was the presence of five carbon resonances (*δ*_C_ 170.9, 170.1, 67.0, 51.3, and 38.1) in **5**. The HMBC correlations from OMe-4′ to C-4′, H-2′ to C-3′/C-4′, and OH-2′ to C-1′ (Fig. 2 and Supplementary Fig. 39–43) indicated **5** had a methyl malate moiety (Supplementary Fig. 44). Its linkage was deduced at C-5 based on the downfield shift of C-5 from *δ*_C_ 76.5 in **8** to *δ*_C_ 90.3 in **5**. Moreover, its configuration was established as L-configuration by a polarimetric analyses for the hydrolyzed product of **5** with optical value of −2.0. Thus, **5** was characterized as (2*'* *S*,8*R*,9*S*,10*R*,13*S*,14*R*,16*S*,17*R*,20*S*,22*R*,24*S*,25*S*)-5-L-methyl malatephysalin D.

Physalin IX (**6**) was isolated as an amorphous powder, and the molecular formula was established as C_28_H_32_O_11_ according to HRESIMS *m/z* 567.1842 [M + Na]^+^ (calcd for C_28_H_32_O_11_Na, 567.1837; Supplementary Fig. 54) and ^13^C NMR data (Supplementary Fig. 48). The ^13^C NMR spectrum observed characteristic resonances at *δ*_C_ 112.6 (C-14), 85.7 (C-15), 82.0 (C-17), 75.8 (C-13), and 47.1 (C-11), indicating that **6** was an unusual 11,15-*cyclo* physalin in which C-15 was an oxy-carbon rather than a ketonic carbon^26^. The long-range correlations from H-11 to C-9/C-10/C-13/C-14/C-15 and OH-15 to C-11/C-15/C-16 in the HMBC spectrum (Supplementary Fig. 49–51) confirmed the above deduction. A comparison of ^1^H and ^13^C NMR data for **6** and physalin R (**15**)^26^ indicated the absence of two carbons [*δ*_C_ 135.5 (C-5) and 123.4 (C-6) in **15**] and the presence of two oxygenated carbons [*δ*_C_ 77.2 (C-5) and 74.1 (C-6)] in **6**. The HMBC correlations from OH-5 to C-5, OH-6 to C-6, and H-6 to C-5/C-8/C-10 (Fig. 2) suggested that two hydroxy groups were linked at C-5 and C-6, respectively. The NOESY correlations of H-4a with Me-19, H-4b with OH-5, OH-6 with Me-19, OH-15 with H-11/H-16, Me-28 with H-16 (Fig. 3 and Supplementary Fig. 52 and 53) revealed an *α*-orientation of OH-5, and the *β*-orientations of OH-6, H-11, and OH-15. Accordingly, **6** was identified as (5*R*,6*R*,8*R*,9*S*,10*R*,11*S*,13*S*,14*R*,15*S*,16*R*,17*R*,20*S*,22*R*,24*S*,25*S*)-11*-*hydro-5,6,15-trihydroxyphysalin R.

### Plausible biogenetic pathway

**Figure 5:**
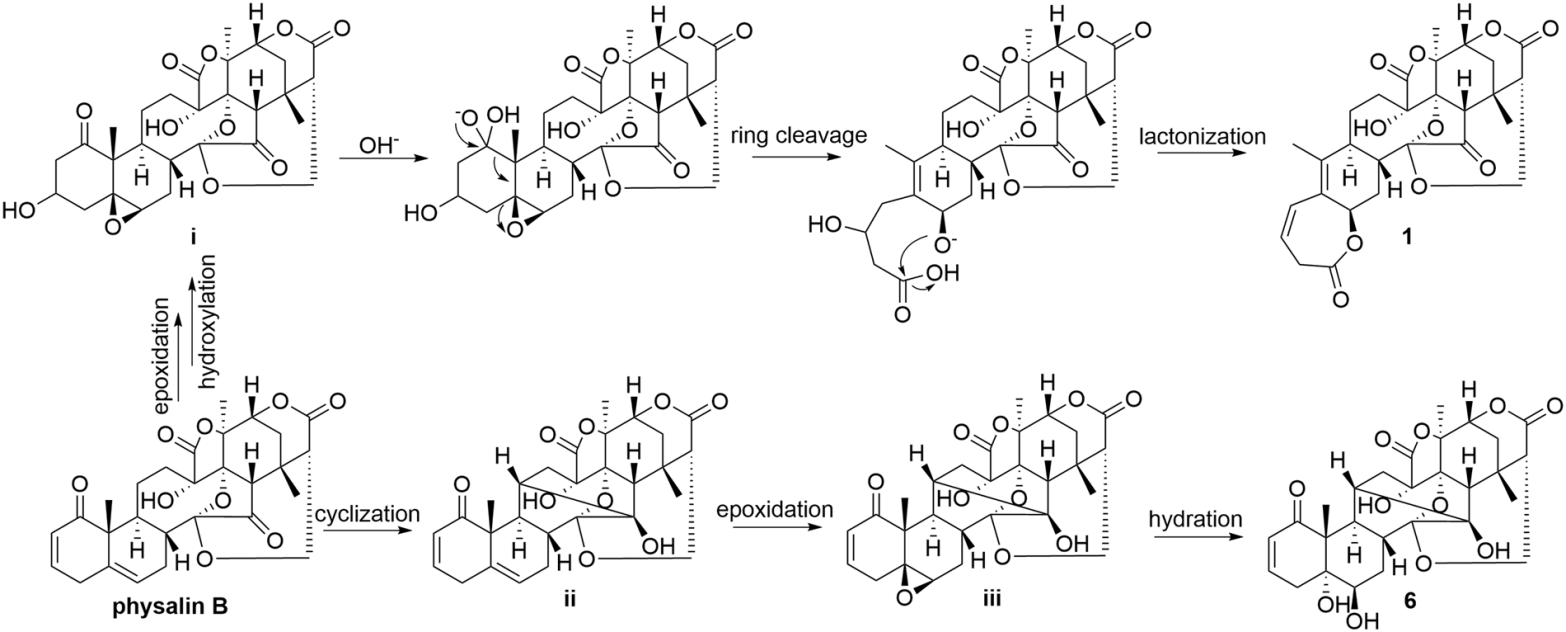
A plausible biogenetic pathway for compounds **1** and **6**.

The discovery of compounds **1** and **6** in the genus *Physalis* is rather uncommon from the viewpoint of chemotaxonomy. Compounds **1** and **6** could have been produced from physalin B, one of the major constituents of *P*. *angulata* (Fig. 5). Epoxidation of physalin B followed by Michael addition reaction from C-3 could give intermediate i. Then nucleophile attack on intermediate i by C-1, followed by ring cleavage between C-1 and C-10, lactonization, and dehydration could yield **1** ^27^. The cyclization of physalin B between C-11 and C-15 could produce intermediate ii as well as physalin R except for the orientations of H-11 and OH-15, which could be further epoxidized and hydrated to give **6**.

### Antiproliferative assay

The isolated compounds were examined for their antiproliferative activities against human prostate cancer cells (C4-2B and 22Rv1), human renal cancer cells (786-O, A-498, and ACHN), human melanoma cancer cells (A375-S2), and their inhibitory effects on NO production induced by LPS in macrophages. In the antiproliferative experiment (Table 3), 5-fluorouracil was used as a positive control drug. Compounds **9** and **10** displayed significant inhibitory effects against all tested cancer cells with IC_50_ values of 0.24 and 3.17 *μ*M, respectively.

**Table 3:** IC_50_ values^*a*^ of tested compounds against human cancer cell lines.

Compound	C4-2B	22Rv1	786-O	A-498	ACHN	A375-S2
	(mean ± SD, *μ*M)	(mean ± SD, *μ*M)	(mean ± SD, *μ*M)	(mean ± SD, *μ*M)	(mean ± SD, *μ*M)	(mean ± SD, *μ*M)
**9**	0.30 ± 0.04	0.36 ± 0.02	0.72 ± 0.11	0.57 ± 0.03	0.96 ± 0.08	3.17 ± 0.17
**10**	0.50 ± 0.03	0.30 ± 0.02	0.24 ± 0.06	0.33 ± 0.02	0.82 ± 0.09	2.61 ± 0.14
5-fluorouracil^*c*^	5.64 ± 0.45	3.83 ± 0.16	>10	8.83 ± 0.88	2.73 ± 0.79	1.91 ± 0.54

^*a*^Results were expressed as IC_50_ values in *μ*M.

^*b*^Compounds **1**–**8** and **11**–**16** were inactive for all cell lines used (IC_50_ > 10 *μ*M).

^*c*^Positive control.

### Inhibitory effects of all the compounds on NO production induced by LPS in macrophages

Nitric oxide (NO) is famous as a cellular signaling molecule, and considered as an important regulator in many physiological mechanisms^38–40^. Pharmacological studies have indicated that inflammation is related to overproduction of NO^41^. The inhibitory effects of all isolated compounds on NO production induced by LPS in macrophages were assayed (Table 4). As shown in Table 4, compounds **9**, **10**, **13**, and **16** exhibited significant inhibitory activities against NO production with IC_50_ values of 0.32–4.03 *μ*M, while compounds **1**, **3**, **4**, and **14** showed moderate inhibitory activities with IC_50_ values of 12.83–34.19 *μ*M. A comparison of the inhibitory efficiency of physalins revealed that the Δ^5,6^ double bond and the proton at C-25 were of pivotal importance. Compound **16** displayed significant inhibitory effect, while **4** showed moderate inhibitory activity, indicating that H-25 could increase inhibitory activity. An analogous case was observed for compounds **8** and **9**, the former showed weak inhibitory effect since the 5-ene unit was hydroxylated. However, compound **3** displayed moderate inhibitory effect, indicating that the configurations of OH-5 and OH-6 could influence inhibitory activity. All isolated compounds were evaluated for their cytotoxic effects against RAW 264.7 macrophages, but did not exhibit any at their effective concentration.

**Table 4:** Inhibitory effects of compounds **1**–**16** on NO production induced by LPS in macrophages.

compound	IC_50_ (mean ± SD, *μ*M)	compound	IC_50_ (mean ± SD, *μ*M)
**1**	19.72 ± 1.16	**10**	0.32 ± 0.14
**2**	>100	**11**	>100
**3**	12.83 ± 0.93	**12**	78.87 ± 5.01
**4**	34.19 ± 2.26	**13**	3.01 ± 0.20
**5**	69.81 ± 4.44	**14**	25.91 ± 1.53
**6**	>100	**15**	70.69 ± 4.93
**7**	>100	**16**	4.03 ± 0.26
**8**	54.06 ± 3.89	hydrocortisone^*a*^	58.79 ± 3.32
**9**	0.88 ± 0.22		

^*a*^Positive control.

## Conclusion

In summary, a 1,10-*seco* physalin, physalin V (**1**), physalin VIII (**5**), a novel 11,15-*cyclo* physalin, physalin IX (**6**) and two other new ones (**2** and **4**) were isolated from the stems and leaves of *P*. *angulata* together with eleven known ones. The absolute configuration of physalin P (**12**) was established by single crystal X-ray crystallography, and it is the first report about the absolute configuration of the neophysalins. To our knowledge, the 1,10-*seco* normal withanolides were previously isolated from *P*. *minmina*, *P*. *peruviana* and *Flos Daturae*, while it is the first report about the presence of 1,10-*seco* physalin in nature and the genus *Physalis*. The inhibitory effects on nitric oxide production and antiproliferative activities against human cancer cells of the isolated compounds were evaluated. Compounds **9** and **10** showed significant antiproliferative activities against all tested human cancer cells. Compounds **9**, **10**, **13**, and **16** showed significant inhibitory activities against NO production. These results indicated that they are promising candidates that could be further researched on and developed as antitumor and anti-inflammatory agents.

## Methods

### General experimental procedures

Optical rotations were recorded on a Perkin-Elmer 241 polarimeter. UV spectra were measured on a Shimadzu UV 2201 spectrophotometer. IR spectra were recorded on a Bruker IFS 55 spectrometer. Bruker AV-400 and AV-600 spectrometers were used in the NMR experiments. Chemical shift values were expressed in *δ* (ppm) using the peak signals of the solvent DMSO-*d*_6_ (*δ*_H_ 2.50 and *δ*_C_ 39.51) as references, and coupling constants (*J* in Hz) were given in parentheses. HRESIMS data were acquired on an Agilent 6210 TOF mass spectrometer. Silica gel GF_254_ prepared for TLC was purchased from Qingdao Marine Chemical Factory (Qingdao, China). Silica gel (200–300 mesh, Qingdao Marine Chemical Factory, Qingdao, China), Sephadex LH-20 (Pharmacia, USA), and octadecyl silica gel (Merck Chemical Company Ltd., German) were used for column chromatography (CC). RP-HPLC separations were conducted using an LC-6AD liquid chromatograph and a SPD-20A UV detector (Shimadzu, Kyoto, Japan) with a RP-C_18_ column (250 × 20 mm, 120 Å, 5 *μ*m, YMC Co. Ltd.).

### Plant material

The stems and leaves of *P*. *angulata* were collected from Nanning, Guangxi Province, China, in July 2013, and identified by Jia-Fu Wei, Guangxi Institute for Food and Drug Control. A voucher specimen (PA-20130826) has been deposited in the herbarium of the Department of Natural Products Chemistry, Shenyang Pharmaceutical University.

### Extraction and isolation

The dried stems and leaves of *P*. *angulata* (9.5 kg) were extracted with 75% EtOH (2 × 2 h × 110 L) and concentrated *in vacuo*. The resulting extracts (1.3 kg) were suspended in H_2_O (5 L), and partitioned successively with petroleum ether (3 × 5 L), EtOAc (3 × 5 L), and *n*-BuOH (3 × 5 L). The EtOAc extracts (116 g) were subjected to silica gel CC (10 × 80 cm) eluted with CH_2_Cl_2_–MeOH (100:1, 80:1, 60:1, 40:1, 20:1, 10:1, 8:1, 5:1, 3:1, 1:1, and 0:1, v/v) to afford compound **8** (500 mg) and six fractions (E1−E6). E3 (35 g) was subjected to silica gel CC (6 × 80 cm) eluted with petroleum ether–acetone (10:1 to 0:1) to produce seven subfractions (E31−E37). E33 (4.0 g) was separated by ODS CC (3 × 50 cm) using a gradient of increasing MeOH in H_2_O (1:9 to 1:0) to yield three subfractions (E331−E333). E331 (2 g) was chromatographed over silica gel CC (2 × 50 cm, CHCl_3_–MeOH, 80:1 to 1:1) and preparative TLC (CH_2_Cl_2_–acetone, 4:1), yielding compound **6** (12 mg). E332 (1.5 g) was separated by silica gel CC (2 × 50 cm, petroleum ether–acetone, 50:1 to 1:1), preparative TLC (CH_2_Cl_2_–acetone, 4:1), and preparative HPLC (60% MeOH–H_2_O, 6 mL min^−1^) to afford compounds **2** (10 mg, *t*_R_ = 24 min) and **14** (21 mg, *t*_R_ = 26 min). Compound **9** (140 mg) was recrystallized from E34 (4.2 g) using MeOH. E36 (3.8 g) was subjected to silica gel CC (5 × 70 cm), eluted with petroleum ether–acetone (80:1 to 1:1), to afford five subfractions (E361−E365). E363 (1.5 g) was subjected to silica gel CC (3.5 × 70 cm) using CH_2_Cl_2_–acetone (80:1 to 4:1) to produce compound **10** (200 mg). E364 (210 mg) was separated by silica gel CC (2 × 50 cm, CH_2_Cl_2_–acetone, 80:1 to 4:1) and preparative TLC (CH_2_Cl_2_–acetone, 4:1) to yield compound **12** (28 mg). E37 (3.2 g) was purified by preparative TLC (CH_2_Cl_2_–EtOAc, 1:1) to give compounds **1** (19 mg) and **11** (25 mg). E4 (15 g) was subjected to silica gel CC (5 × 70 cm), eluted with CHCl_3_–acetone (80:1 to 1:1), to afford five subfractions (E41−E45). E43 (1.0 g) was chromatographed over ODS CC (3 × 50 cm, MeOH–H_2_O, 1:9 to 1:0) and preparative HPLC (60% MeOH–H_2_O) to give compound **7** (10 mg, *t*_R_ = 24 min). E45 (4 g) was separated by an ODS column (3 × 50 cm, MeOH–H_2_O, 1:9 to 1:0) and preparative TLC (CH_2_Cl_2_–acetone, 2:1), yielding compound **3** (50 mg) and an impure subfraction, which was further purified by preparative HPLC (65% MeOH–H_2_O) to afford compounds **5** (8 mg, *t*_R_ = 20 min) and **15** (10 mg, *t*_R_ = 23 min). E6 (7 g) was subjected to silica gel CC (5 × 70 cm) eluted with CHCl_3_–acetone (50:1 to 1:1) to afford four subfractions (E61−E64). E61 (900 mg) was separated by Sephadex LH-20 CC (3 × 80 cm, MeOH) and preparative HPLC (60% MeOH–H_2_O) to produce compound **13** (5 mg, *t*_R_ = 16 min). E64 (1.5 g) was subjected to ODS CC (3 × 50 cm, MeOH–H_2_O, 1:9 to 1:0) and preparative TLC (CH_2_Cl_2_–acetone, 10:1), yielding compounds **4** (8 mg) and **16** (28 mg).

### Spectroscopic data of 1–6

Physalin V (**1**): amorphous powder; ${[\alpha ]}_{{\rm{D}}}^{25}$ −12.0 (*c* 0.05, MeOH); UV (MeOH) *λ*_max_ (log *ε*) 234 (4.0) nm; IR (KBr) *ν*_max_ 3400, 2921, 2850, 1782, 1765, 1728, 1646, 1385, 1143 cm^−1^; ^1^H (600 MHz, DMSO-*d*_6_) and ^13^C NMR (150 MHz, DMSO-*d*_6_) data, see Table 1; HRESIMS *m/z* 549.1750 [M + Na]^+^ (calcd for C_28_H_30_O_10_Na, 549.1737).

**Table 1:** ^1^H and ^13^C NMR data of compounds **1**−**3**.

*no*.	1^*a*^		2^*b*^		3^*b*^	
	*δ*_C_	*δ*_H_ (*J* in Hz)	*δ*_C_	*δ*_H_ (*J* in Hz)	*δ*_C_	*δ*_H_ (*J* in Hz)
1	171.79		209.6		202.7	
2	35.6	4.09, br d (17.6)	39.6	3.42, d (20.0)	127.3	5.79, dd (10.0, 2.0)
		3.02, dd (17.6, 9.3)		2.61, dd (20.0, 4.3)		
3	118.3	5.49, ddd (11.2, 9.3, 2.0)	122.6	5.67, m	143.4	6.69, ddd (10.0, 5.1, 2.0)
4	128.8	6.49, dd (11.2, 2.0)	128.0	6.07, br d (11.2)	29.7	2.60, dt (20.2, 2.0)
						2.33, dd (20.2, 5.1)
5	125.7		140.4		77.4	
6	71.2	5.25, br s	126.6	5.70, br d (4.8)	70.4	3.58, m
7	26.1	2.29, br d (14.3)	26.1	2.44, dd (14.3, 4.8)	29.8	2.08, m
		1.36, td (14.3, 3.9)		2.07, m		1.34, m
8	40.0	2.09, td (9.4, 3.9)	40.5	2.06, m	41.2	2.01, td (12.4, 4.5)
9	38.4	3.38, t (9.4)	32.5	2.95, dd (10.4, 7.4)	33.4	2.98, br t (12.4)
10	141.0		55.3		55.7	
11	23.7	2.19, m	23.7	1.36, m	21.1	1.33, m
		1.43, m		1.08, m		0.80, m
12	24.1	2.19, m	28.8	2.15, m	24.3	1.76, m
		1.43, m		1.86, dd (15.5, 6.5)		1.27, m
13	80.5		79.3		78.3	
14	105.2		101.0		105.9	
15	209.0		215.2		208.9	
16	53.9	2.86, s	51.3	2.78, s	54.1	2.75, s
17	78.8		82.3		80.4	
18	171.80		172.3		171.6	
19	17.1	1.82, s	17.8	1.05, s	8.5	0.81, s
20	80.3		82.8		80.2	
21	21.4	1.83, s	20.7	1.75, s	21.4	1.78, s
22	76.4	4.61, t (2.6)	76.7	4.42, br d (5.3)	76.3	4.56, t (2.9)
23	31.2	2.13, dd (14.6, 2.6)	26.9	2.71, dd (14.3, 5.3)	31.2	2.09, m
		1.94, br d (14.6)		1.38, m		1.89, m
24	30.5		41.1		30.5	
25	49.2	2.96, d (4.4)	72.6		49.2	2.90, d (4.4)
26	167.2		169.8		167.2	
27	61.2	4.31, dd (13.4, 4.4)	19.4	1.36, s	60.8	4.25, dd (13.4, 4.4)
		3.66, br d (13.4)				3.57, dd (13.4, 4.4)
28	24.3	1.17, s	22.4	1.43, s	24.4	1.13, s
OH-5						4.40, s
OH-6						4.53, d (5.0)
OH-13		6.86, s		6.08, s		6.36, s
OH-14				6.43, s		
OH-25				5.93, s		

^*a*1^H NMR spectra recorded at 600 MHz, ^13^C NMR spectra recorded at 150 MHz, DMSO-*d*_6_.

^*b*1^H NMR spectra recorded at 400 MHz, ^13^C NMR spectra recorded at 100 MHz, DMSO-*d*_6_.

Physalin VI (**2**): amorphous powder; ${[\alpha ]}_{{\rm{D}}}^{25}$ −64.0 (*c* 0.05, MeOH); UV (MeOH) *λ*_max_ (log *ε*) 216 (3.8) nm; IR (KBr) *ν*_max_ 3397, 2921, 2850, 1716, 1646, 1467, 1384, 1111 cm^−1^; ^1^H (400 MHz, DMSO-*d*_6_) and ^13^C NMR (100 MHz, DMSO-*d*_6_) data, see Table 1; HRESIMS *m/z* 527.1918 [M−H]^−^ (calcd for C_28_H_31_O_10_, 527.1917).

Physalin D_1_ (**3**): amorphous powder; ${[\alpha ]}_{{\rm{D}}}^{25}$ –43.6 (*c* 0.05, MeOH); UV (MeOH) *λ*_max_ (log *ε*) 220 (3.9) nm; IR (KBr) *ν*_max_ 3396, 2921, 2850, 1765, 1734, 1648, 1468, 1384, 1134 cm^−1^; ^1^H (400 MHz, DMSO-*d*_6_) and ^13^C NMR (100 MHz, DMSO-*d*_6_) data, see Table 1; HRESIMS *m/z* 543.1870 [M − H]^−^ (calcd for C_28_H_31_O_11_, 543.1866).

Physalin VII (**4**): amorphous powder; ${[\alpha ]}_{{\rm{D}}}^{25}$ (*c* 0.055, MeOH) −138.2; UV (MeOH) *λ*_max_ (log *ε*) 218 (4.0) nm; IR (KBr) *ν*_max_ 3431, 2920, 2850, 1767, 1740, 1697, 1645, 1465, 1384, 1138 cm^−1^; ^1^H (600 MHz, DMSO-*d*_6_) and ^13^C NMR (150 MHz, DMSO-*d*_6_) data, see Table 2; HRESIMS *m/z* 525.1769 [M − H]^−^ (calcd for C_28_H_29_O_10_, 525.1761).

**Table 2:** ^1^H and ^13^C NMR data of compounds **4**−**6**.

*no*.	4^*a*^		5^*b*^		6^*a*^	
	*δ*_C_	*δ*_H_ (*J* in Hz)	*δ*_C_	*δ*_H_ (*J* in Hz)	*δ*_C_	*δ*_H_ (*J* in Hz)
1	209.8		203.1		203.9	
2	39.6	3.42, d (20.0)	126.7	5.75, dd (10.1, 2.2)	127.3	5.70, dd (10.0, 2.0)
		2.62, dd (20.0, 4.7)				
3	122.6	5.66, m	142.6	6.68, ddd (10.1, 5.1, 2.2)	143.9	6.70, ddd (10.0, 5.3, 2.0)
4	128.0	6.06, dd (9.5, 2.1)	28.6	3.27, dd (20.6, 5.1)	34.8	3.03, dt (19.5, 2.0)
				3.11, dt (20.6, 2.2)		2.06, dd (19.5, 5.3)
5	140.4		90.3		77.2	
6	125.7	5.67, m	65.3	4.54, t (3.6)	74.1	3.52, br s
7	24.8	2.33, dd (14.5, 3.8)	26.8	1.89, dt (14.1, 3.6)	28.9	1.85, m
		2.03, m		1.39, td (14.1, 3.6)		1.52, dt (12.5, 2.1)
8	38.5	2.10, td (11.1, 4.3)	37.8	2.22, td (12.2, 3.6)	40.0	2.41, td (12.5, 2.1)
9	31.7	3.08, br t (11.1)	29.9	3.32, td (12.2, 5.4)	41.0	2.75, dd (12.7, 6.7)
10	54.6		53.5		54.1	
11	24.6	1.57, m	24.3	1.85, m	47.1	2.23, t (6.0)
		1.03, m		1.01, m		
12	25.1	2.34, m	25.8	2.16, m	31.3	2.55, br d (14.8)
		1.42, dd (15.8, 9.9)		1.46, dd (16.0, 9.7)		1.87, m
13	78.3		78.7		75.8	
14	106.2		106.5		112.6	
15	209.0		209.7		85.7	
16	54.2	2.98, s	54.0	2.82, s	49.8	1.79, s
17	80.4		80.8		82.0	
18	171.5		171.6		173.9	
19	18.4	1.21, s	13.2	1.18, s	13.9	1.17, s
20	79.5		80.5		81.9	
21	21.8	1.80, s	21.2	1.81, s	20.5	1.61, s
22	76.6	4.59, dd (3.5, 2.1)	76.3	4.56, t (2.9)	75.9	4.39, br t (2.6)
23	28.0	2.35, dt (14.2, 3.5)	31.3	2.10, dd (14.5, 2.9)	34.7	1.89, m
		1.76, dd (14.2, 2.1)		1.93, dd (14.5, 2.9)		1.77, m
24	35.5		30.5		31.4	
25	73.6		49.4	2.89, d (4.2)	50.2	2.69, d (3.8)
26	168.4		167.3		168.9	
27	64.6	3.95, br d (12.7)	60.6	4.25, dd (13.4, 4.2)	59.9	4.70, br d (11.8)
		3.37, br d (12.7)		3.59, br d (13.4)		4.02, dd (11.8, 3.8)
28	18.9	1.12, s	24.4	1.16, s	28.5	1.34, s
1′			171.1			
2′			67.0	4.13, ddd (7.8, 5.1, 3.6)		
3′			38.1	2.52, dd (16.0, 3.6)		
				2.36, dd (16.0, 7.8)		
4′			170.1			
OMe-4′			51.3	3.52, s		
OH-2′				5.52, d (5.3)		
OH-5						4.15, s
OH-6				5.41, d (4.9)		4.88, d (4.0)
OH-13		6.37, s		6.03, s		5.95, s
OH-15						5.50, s
OH-25		6.45, s				

^*a*1^H NMR spectra recorded at 600 MHz, ^13^C NMR spectra recorded at 150 MHz, DMSO-*d*_6_.

^*b*1^H NMR spectra recorded at 600 MHz, ^13^C NMR spectra recorded at 100 MHz, DMSO-*d*_6_.

Physalin VIII (**5**): amorphous powder; ${[\alpha ]}_{{\rm{D}}}^{25}$ −76.4 (*c* 0.055, MeOH); UV (MeOH) *λ*_max_ (log *ε*) 216 (3.5) nm; IR (KBr) *ν*_max_ 3442, 2921, 2850, 1791, 1753, 1729, 1687, 1647, 1441, 1383, 1260, 1167 cm^−1^; ^1^H (600 MHz, DMSO-*d*_6_) and ^13^C NMR (100 MHz, DMSO-*d*_6_) data, see Table 2; HRESIMS *m/z* 673.2127 [M − H]^−^ (calcd for C_33_H_37_O_15_, 673.2132).

Physalin IX (6): amorphous powder; ${[\alpha ]}_{{\rm{D}}}^{25}$ –53.0 (*c* 0.055, MeOH); UV (MeOH) *λ*_max_ (log *ε*) 218 (3.7) nm; ^1^H (600 MHz, DMSO-*d*_6_) and ^13^C NMR (150 MHz, DMSO-*d*_6_) data, see Table 2; HRESIMS *m/z* 567.1837 [M + Na]^+^ (calcd for C_28_H_32_O_11_Na, 567.1842).

### X-ray crystal structure determination of compound 12

The data were collected on an Xcalibur, Eos, Gemini diffractometer using monochromatized Cu K*α* radiation. The structure was solved by direct methods using SHELXL. Crystallographic data have been hosted in the Cambridge Crystallographic Data Centre (CCDC number 1465139). Copies of the data can be obtained, free of charge, from the CCDC website (www.ccdc.cam.ac.uk). Crystal Data: C_29_H_35.30334_O_11.65167_, *M* = 570.30, orthorhombic, size 0.16 × 0.09 × 0.05 mm^3^, *a* = 7.65075(19) Å, *b* = 17.3380(6) Å, *c* = 19.8734(5) Å, *α* = *β* = *γ* = 90*°*, *V* = 2636.18(13) Å^3^, *T* = 103.2, space group *P*2_1_2_1_2_1_ (no. 19), *Z* = 4, *μ*(Cu K*α*) = 0.937, completeness *θ*_max_ = 100.0%, *F*(000) = 1210, 2*θ* range for data collection from 6.766 to 143.782*°*, 9459 reflections measured, 5071 unique (*R*_int_ = 0.0293) which were used in all calculations. The final *wR*(*F*_2_) was 0.0974 (all data). The Flack parameter was 0.04(11). The largest difference peak and hole were 0.293 and −0.200 e Å^−3^.

### Optical rotation analysis for hydrolyzed product of compound 5

Compound **5** (3 mg) and hydrochloric acid (2 M, 4 mL) were added into the flask (10 mL) with cover, and stirred at 90 °C for 3 h. The reaction mixture was extracted thrice with CHCl_3_, then the water layer was freeze-dried *in vacuo* to afford the residue. The hydrolyzed product (0.3 mg), purified over a Sephedax LH-20 column from the residue using CHCl_3_-MeOH (1:1), was analyzed by a Perkin-Elmer 241 polarimeter (Perkin-Elmer, Waltham, MA, USA) and an API3200 mass spectrometer (AB SCIEX, Framingham, MA, USA).

### Antiproliferative assay

Compounds were evaluated by the MTT method for antiproliferative activities against human prostate cancer cells (C4-2B and 22Rvl), human renal carcinoma cells (786-O, A-498, and ACHN), and human melanoma cells (A375-S2)^42^. All these cells were incubated in RPMI-1640 or EMEM medium with 10% fetal bovine serum at a humidified atmosphere (5% CO_2_, 37 °C). Cells (1 × 10^4^ cells/well) were added into the 96-well plates for 12 h before drug addition. The test compounds with various concentrations were added into the 96-well plates, then incubated for 48 h. 5-Fluorouracil was used as the positive control, and every assay was repeated three times. Cell viability was evaluated by the 3-(4,5-dimethylthiazol-2-yl)-2,5-diphenyltetrazolium bromide (MTT) reduction assay.

### NO production bioassay

All compounds were assayed for the inhibition of NO production according to the Griess method^43, 44^. 1 × 10^6^ Cells/well of RAW 264.7 cells were added into the 96-well plates, and incubated at 37 °C for 24 h by the stimulation of LPS (1 *μ*g/mL) with or without test compounds. After the addition of Griess reagent [0.1% *N*-(1-naphthyl)-ethylenediamine (50 *μ*L); 1% sulfanilamide in 5% H_3_PO_4_ (50 *μ*L)], absorbance (540 nm) was recorded by using a microplate reader. The standard curve was used to calculate the NO concentrations and inhibitory rates.

## Electronic supplementary material

Supplemmentary information comprises 1D and 2D NMR, and HRESIMS spectra for compounds 1−6.
