# Supplementary material for: Physalins V-IX, 16,24-cyclo-13,14-seco withanolides from Physalis angulata and their antiproliferative and anti-inflammatory activities
Source: Sci Rep. 2017 Jun 22;7:4057. doi: 10.1038/s41598-017-03849-9 (PMC5481415; doi:10.1038/s41598-017-03849-9)
Supplement: Supplementary file 1 — Supplemmentary information comprises 1D and 2D NMR, and HRESIMS spectra for compounds 1−6. [file 41598_2017_3849_MOESM1_ESM.pdf]

Supplementary information

Physalins V-IX, 16,24-*cyclo*-13,14-*seco* withanolides from *Physalis angulata* and their antiproliferative and anti-inflammatory activities

Cheng-Peng Sun[1,2], Chong-Yue Qiu[2], Feng Zhao[3], Ning Kang[1],

Li-Xia Chen[2]*, Feng Qiu[1,2]*

List of supplementary information

Supplementary Figure S1. ^{1}H NMR spectrum of **1** (600 MHz, DMSO-d_6)

Supplementary Figure S2. ^{13}C NMR spectrum of **1** (150 MHz, DMSO-d_6)

Supplementary Figure S3. HSQC spectrum of **1** (600 MHz, DMSO-d_6)

Supplementary Figure S4. HMBC spectrum of **1** (600 MHz, DMSO-d_6)

Supplementary Figure S5. Selected HMBC spectrum of **1** (600 MHz, DMSO-d_6)

Supplementary Figure S6. NOESY spectrum of **1** (600 MHz, DMSO-d_6)

Supplementary Figure S7. Selected NOESY spectrum of **1** (600 MHz, DMSO-d_6)

Supplementary Figure S8. HRESIMS spectrum of **1**

Supplementary Figure S9. IR spectrum of **1**

Supplementary Figure S10. ^{1}H NMR spectrum of **2** (400 MHz, DMSO-d_6)

Supplementary Figure S11. ^{13}C NMR spectrum of **2** (100 MHz, DMSO-d_6)

Supplementary Figure S12. HSQC spectrum of **2** (600 MHz, DMSO-d_6)

Supplementary Figure S13. HMBC spectrum of **2** (600 MHz, DMSO-d_6)

Supplementary Figure S14. Selected HMBC spectrum of **2** (600 MHz, DMSO-d_6)

Supplementary Figure S15. NOESY spectrum of **2** (600 MHz, DMSO-d_6)

Supplementary Figure S16. Selected NOESY spectrum of **2** (600 MHz, DMSO-d_6)

Supplementary Figure S17. HRESIMS spectrum of **2**

Supplementary Figure S18. IR spectrum of **2**

Supplementary Figure S19. ^{1}H NMR spectrum of **3** (400 MHz, DMSO-d_6)

Supplementary Figure S20. ^{13}C NMR spectrum of **3** (100 MHz, DMSO-d_6)

Supplementary Figure S21. HSQC spectrum of **3** (600 MHz, DMSO-d_6)

Supplementary Figure S22. HMBC spectrum of **3** (600 MHz, DMSO-d_6)

Supplementary Figure S23. Selected HMBC spectrum of **3** (600 MHz, DMSO-d_6)

Supplementary Figure S24. NOESY spectrum of **3** (600 MHz, DMSO-d_6)

Supplementary Figure S25. Selected NOESY spectrum of **3** (600 MHz, DMSO-d_6)

Supplementary Figure S26. HRESIMS spectrum of **3**

Supplementary Figure S27. IR spectrum of **3**

Supplementary Figure S28. ^{1}H NMR spectrum of **4** (600 MHz, DMSO-d_6)

Supplementary Figure S29. ^{13}C NMR spectrum of **4** (150 MHz, DMSO-d_6)

Supplementary Figure S30. HSQC spectrum of **4** (600 MHz, DMSO-d_6)

Supplementary Figure S31. HMBC spectrum of **4** (600 MHz, DMSO-d_6)

Supplementary Figure S32. Selected HMBC spectrum of **4** (600 MHz, DMSO-d_6)

Supplementary Figure S33. NOESY spectrum of **4** (600 MHz, DMSO-d_6)

Supplementary Figure S34. Selected NOESY spectrum of **4** (600 MHz, DMSO-d_6)

Supplementary Figure S35. HRESIMS spectrum of **4**

Supplementary Figure S36. IR spectrum of **4**

Supplementary Figure S37. ^{1}H NMR spectrum of **5** (400 MHz, DMSO-d_6)

Supplementary Figure S38. ^{13}C NMR spectrum of **5** (100 MHz, DMSO-d_6)

Supplementary Figure S39. HSQC spectrum of **5** (600 MHz, DMSO-d_6)

Supplementary Figure S40. HMBC spectrum of **5** (600 MHz, DMSO-d_6)

Supplementary Figure S41. Selected HMBC spectrum of **5** (600 MHz, DMSO-d_6)

Supplementary Figure S42. NOESY spectrum of **5** (600 MHz, DMSO-d_6)

Supplementary Figure S43. Selected NOESY spectrum of **5** (600 MHz, DMSO-d_6)

Supplementary Figure S44. MS spectrum (A, negative mode; B, positive mode) of the hydrolyzed product for **5**

Supplementary Figure S45. HRESIMS spectrum of **5**

Supplementary Figure S46. IR spectrum of **5**

Supplementary Figure S47. ^{1}H NMR spectrum of **6** (600 MHz, DMSO-d_6)

Supplementary Figure S48. ^{13}C NMR spectrum of **6** (150 MHz, DMSO-d_6)

Supplementary Figure S49. HSQC spectrum of **6** (600 MHz, DMSO-d_6)

Supplementary Figure S50. HMBC spectrum of **6** (600 MHz, DMSO-d_6)

Supplementary Figure S51. Selected HMBC spectrum of **6** (600 MHz, DMSO-d_6)

Supplementary Figure S52. NOESY spectrum of **6** (600 MHz, DMSO-d_6)

Supplementary Figure S53. Selected NOESY spectrum of **6** (600 MHz, DMSO-d_6)

Supplementary Figure S54. HRESIMS spectrum of **6**

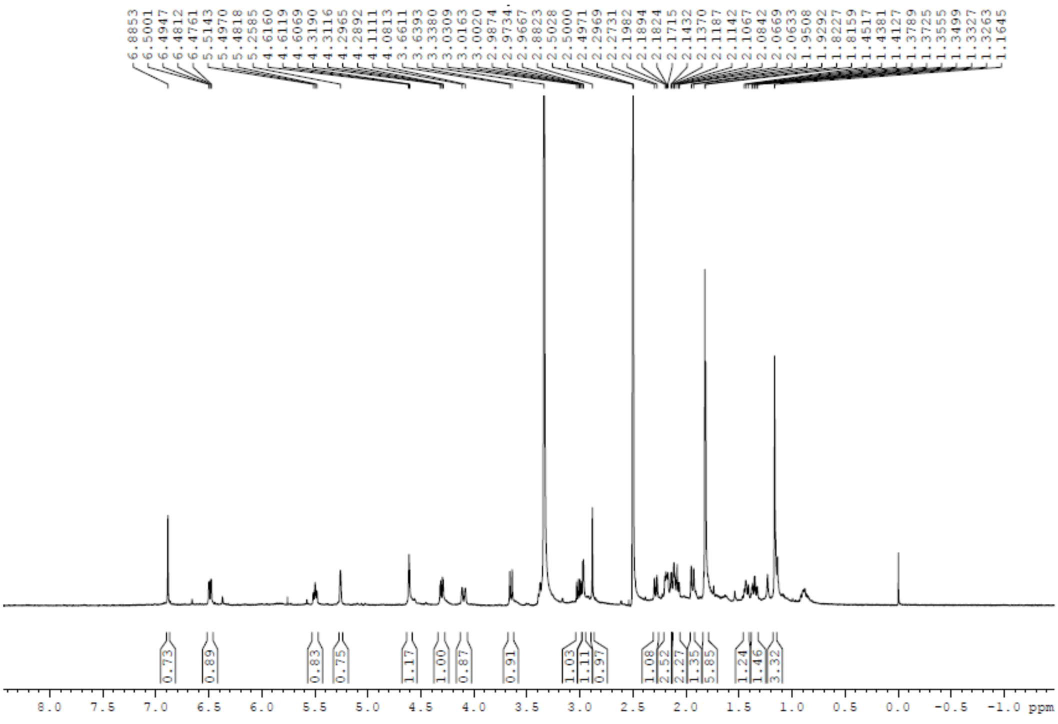

Supplementary Figure S1. ^{1}H NMR spectrum of **1** (600 MHz, DMSO-d_6)

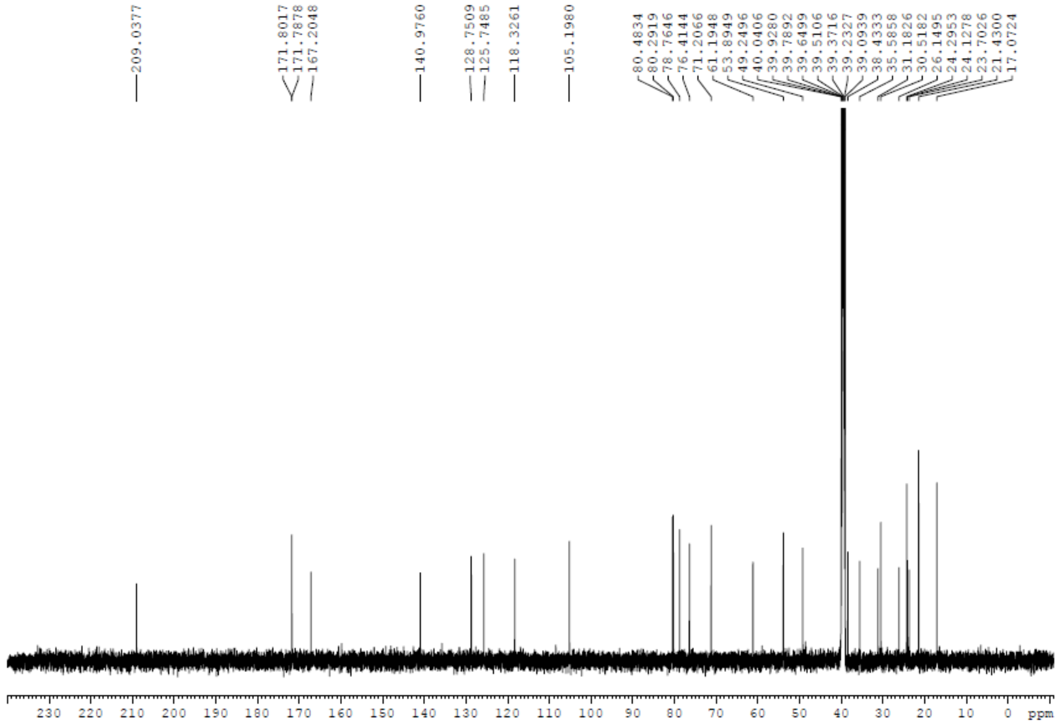

Supplementary Figure S2. ^{13}C NMR spectrum of **1** (150 MHz, DMSO-d_6)

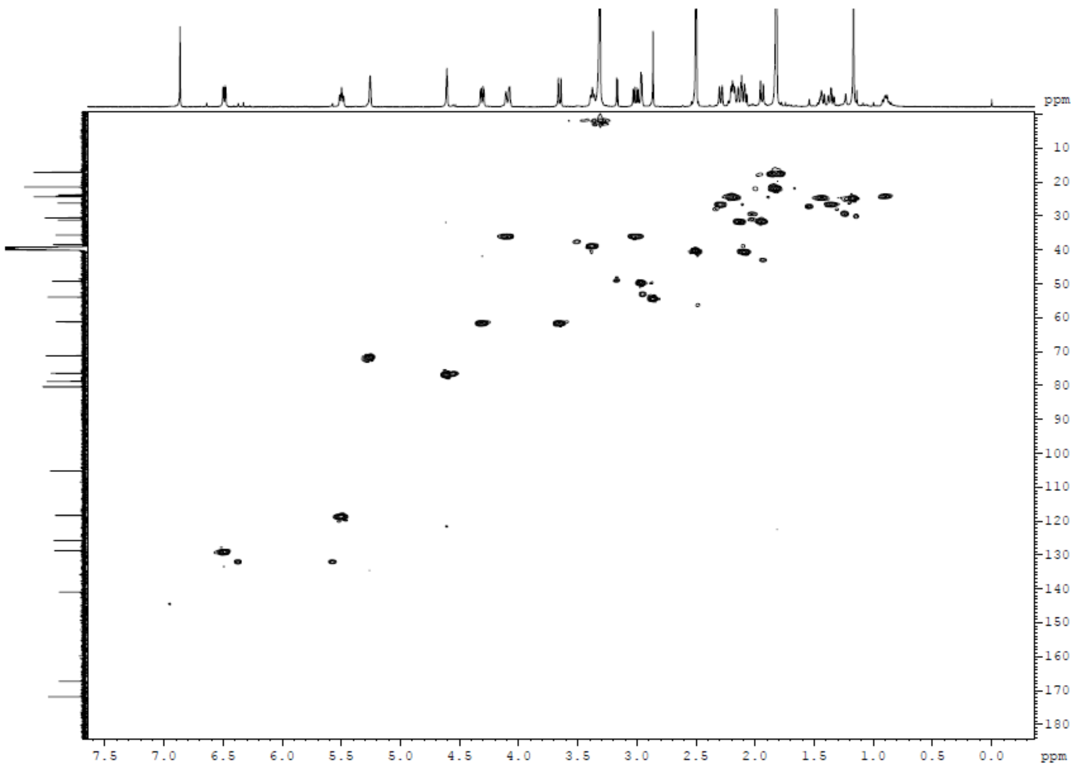

Supplementary Figure S3. HSQC spectrum of **1** (600 MHz, DMSO-d_6)

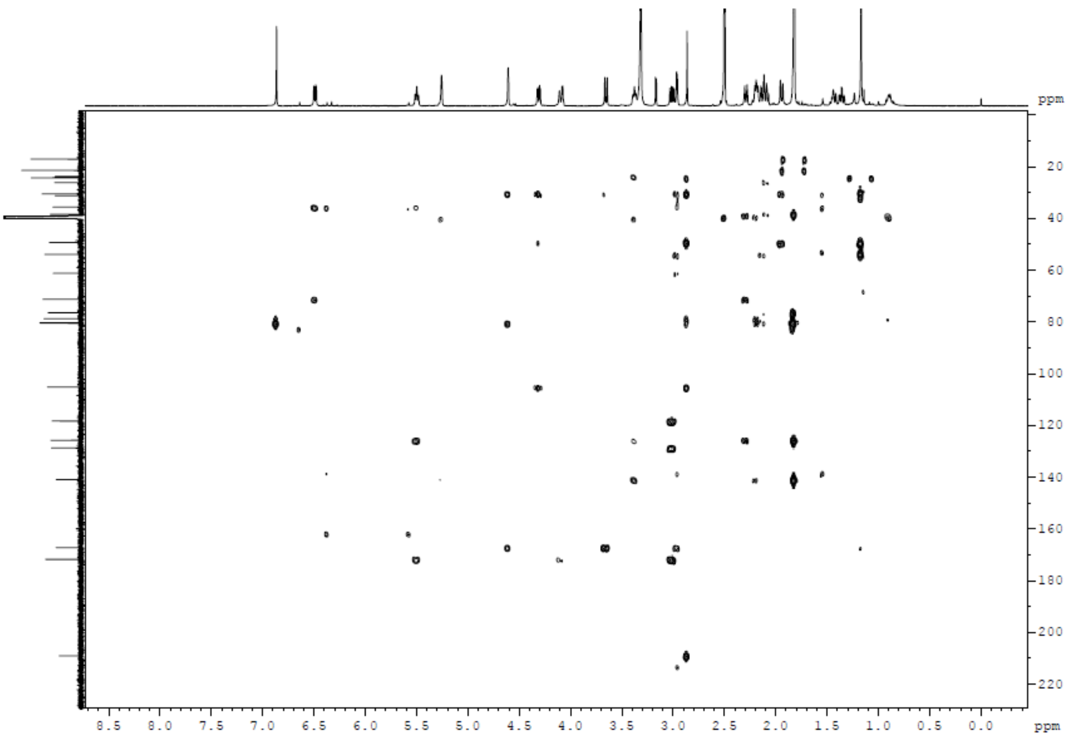

Supplementary Figure S4. HMBC spectrum of **1** (600 MHz, DMSO-d_6)

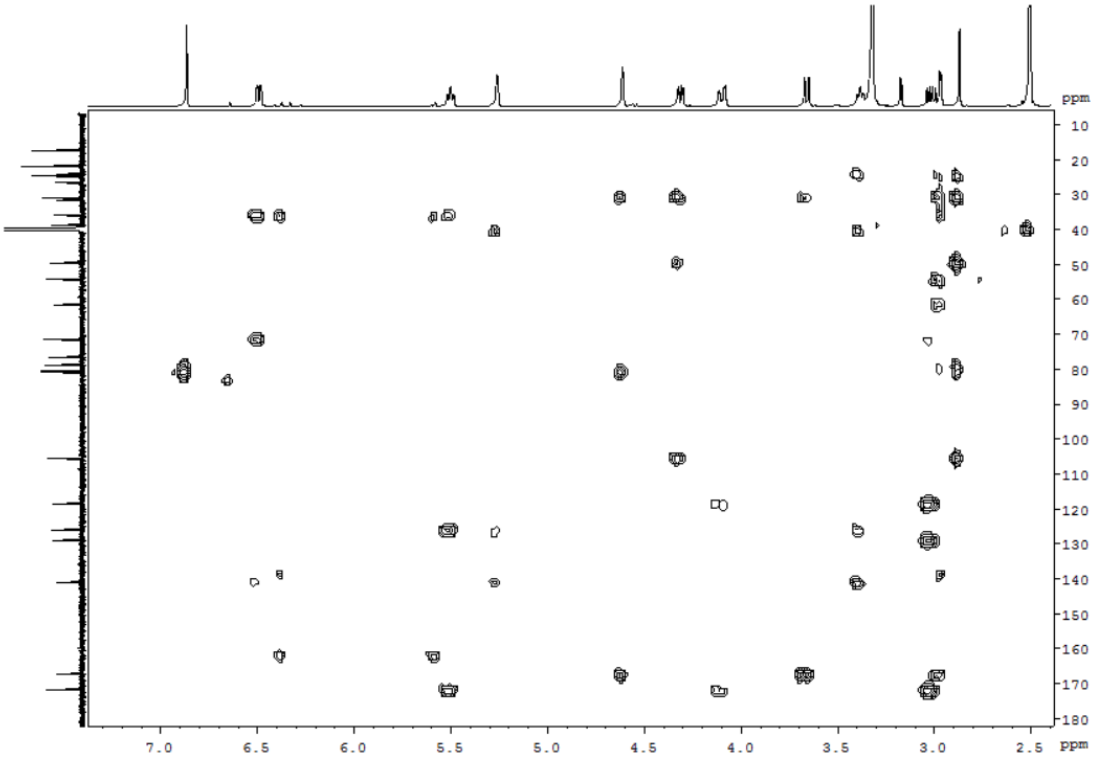

Supplementary Figure S5. Selected HMBC spectrum of **1** (600 MHz, DMSO-d_6)

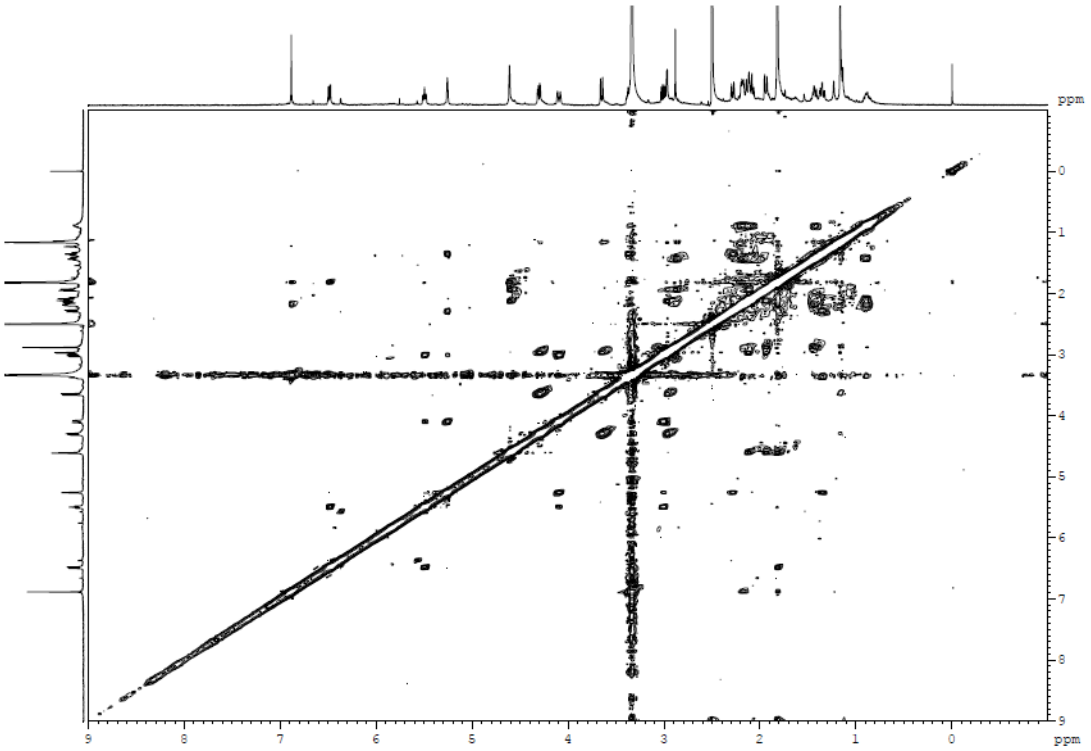

Supplementary Figure S6. NOESY spectrum of **1** (600 MHz, DMSO-d_6)

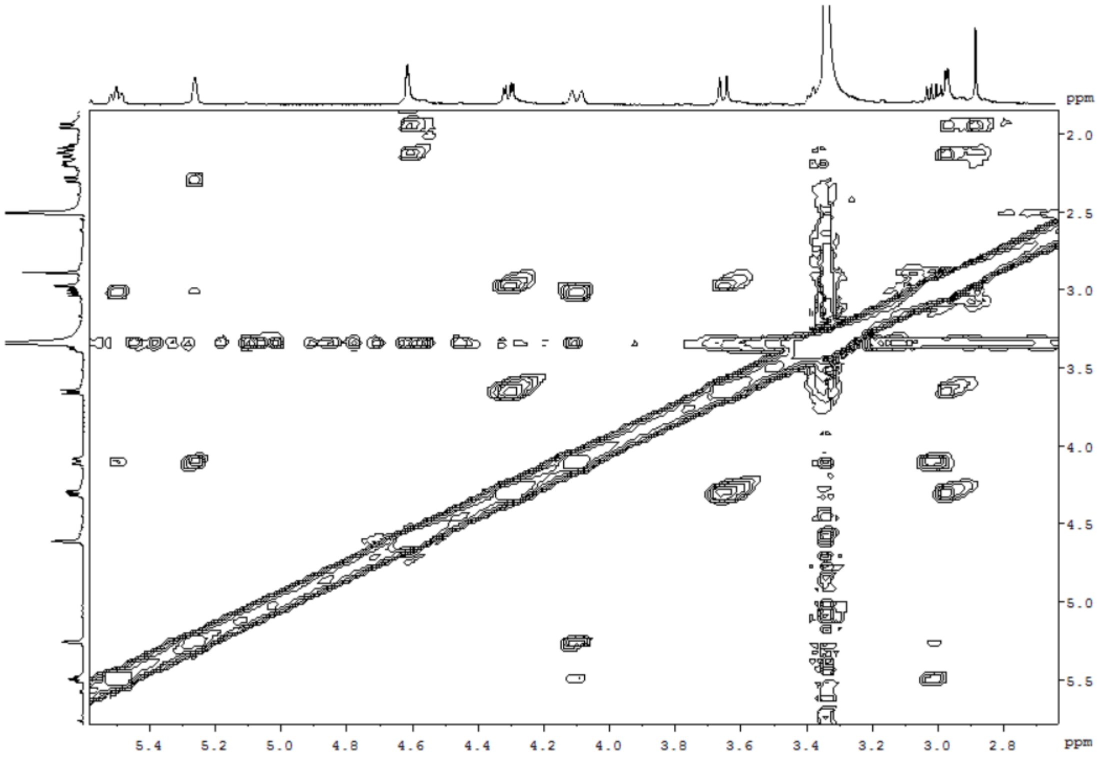

Supplementary Figure S7. Selected NOESY spectrum of **1** (600 MHz, DMSO-

User Chromatograms

User Spectra

Fragmentor Voltage	Collision Energy	Ionization Mode
175	0	ESI

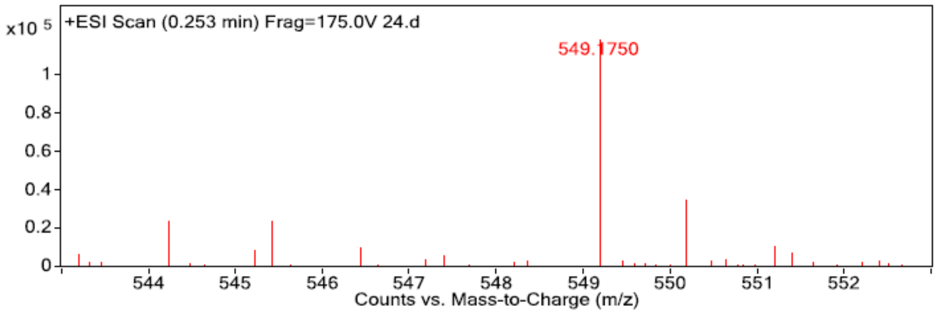

Formula Calculator Results

Formula	Best	Mass	Tgt Mass	Diff (ppm)	Ion Species	Score
C28 H30 O10	TRUE	526.1857	526.1839	-3.52	C28 H30 Na O10	89.71

--- End Of Report ---

Supplementary Figure S8. HRESIMS spectrum of **1**

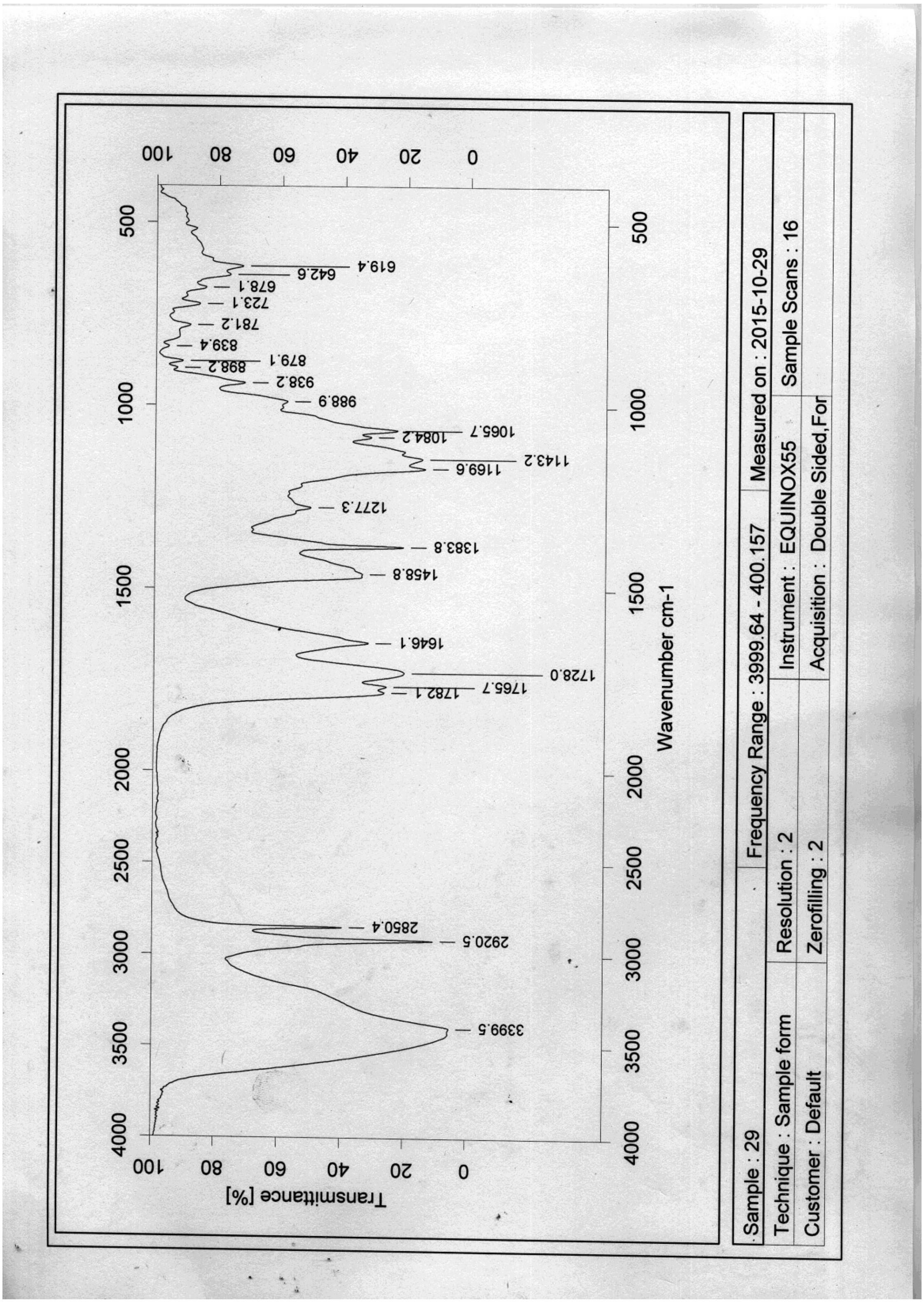

Supplementary Figure S9. IR spectrum of **1**

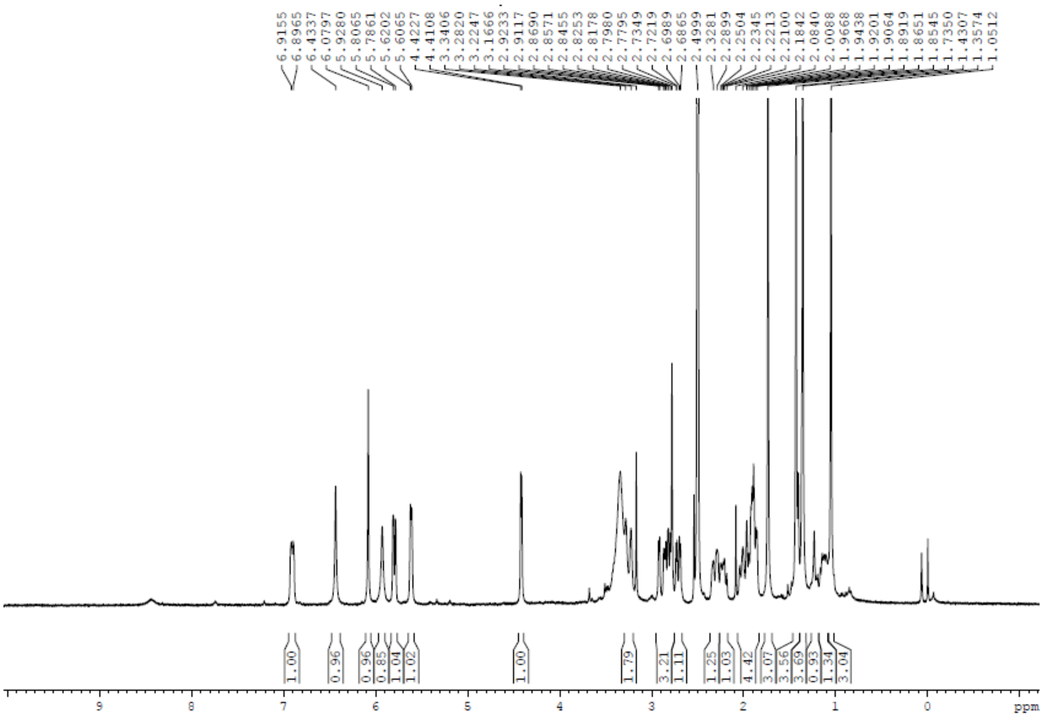

Supplementary Figure S10. ^{1}H NMR spectrum of **2** (400 MHz, DMSO-d_6)

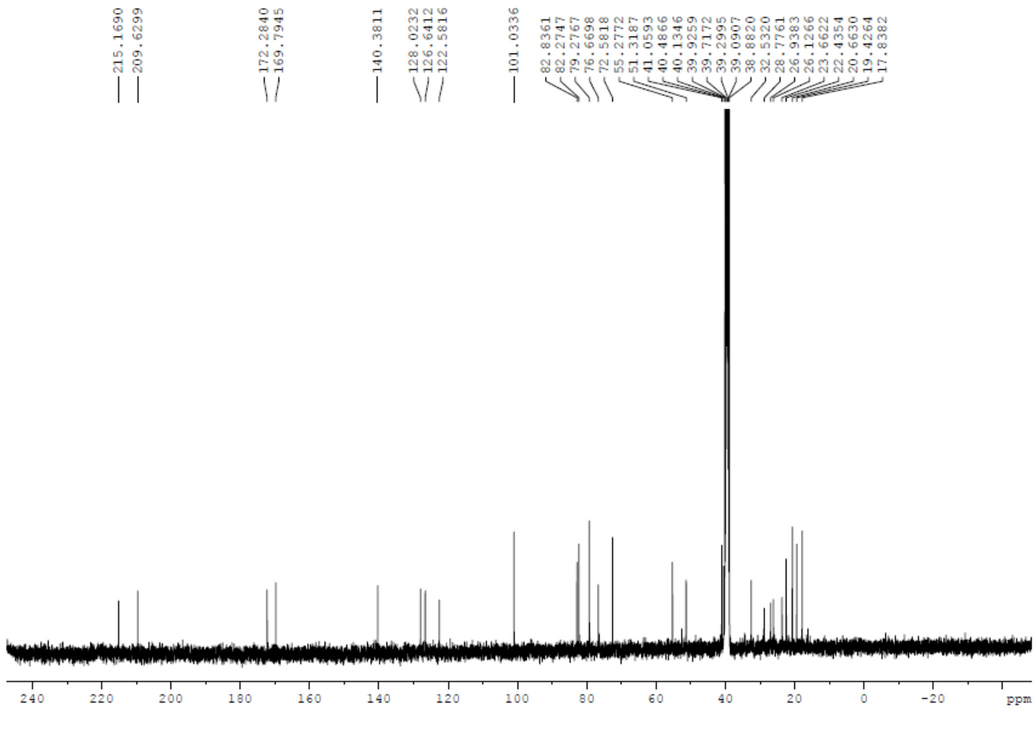

Supplementary Figure S11. ^{13}C NMR spectrum of **2** (100 MHz, DMSO-d_6)

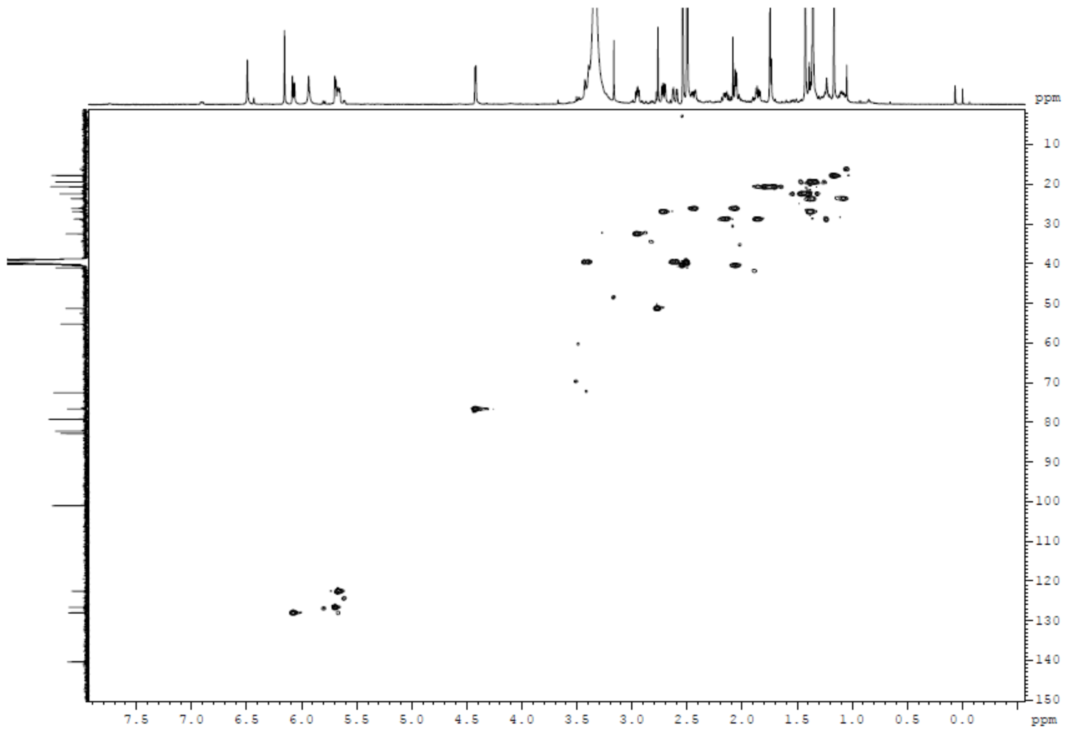

Supplementary Figure S12. HSQC spectrum of **2** (600 MHz, DMSO-d_6)

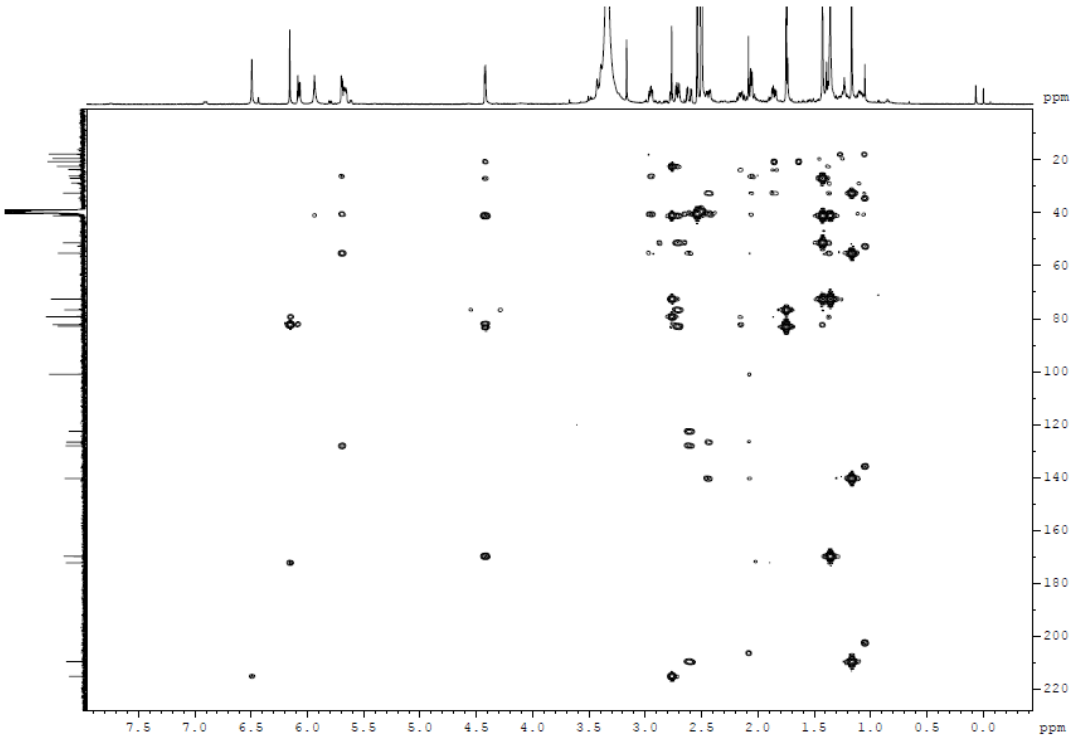

Supplementary Figure S13. HMBC spectrum of **2** (600 MHz, DMSO-d_6)

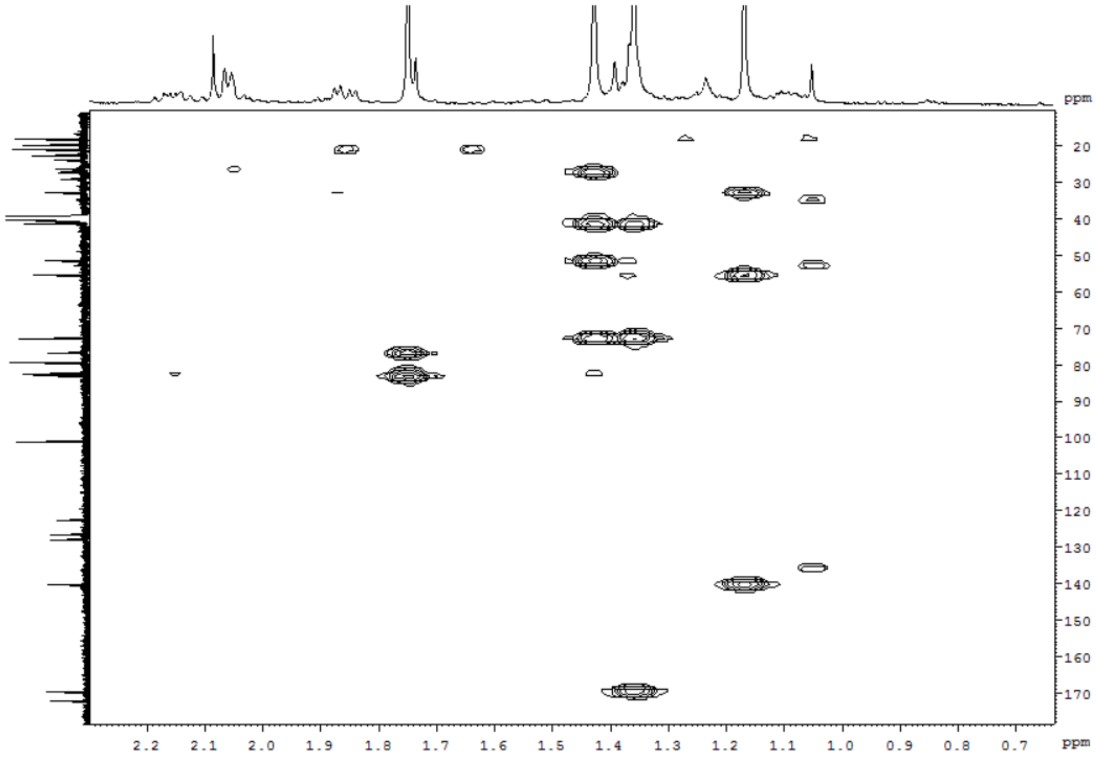

Supplementary Figure S14. Selected HMBC spectrum of **2** (600 MHz, DMSO-d_6)

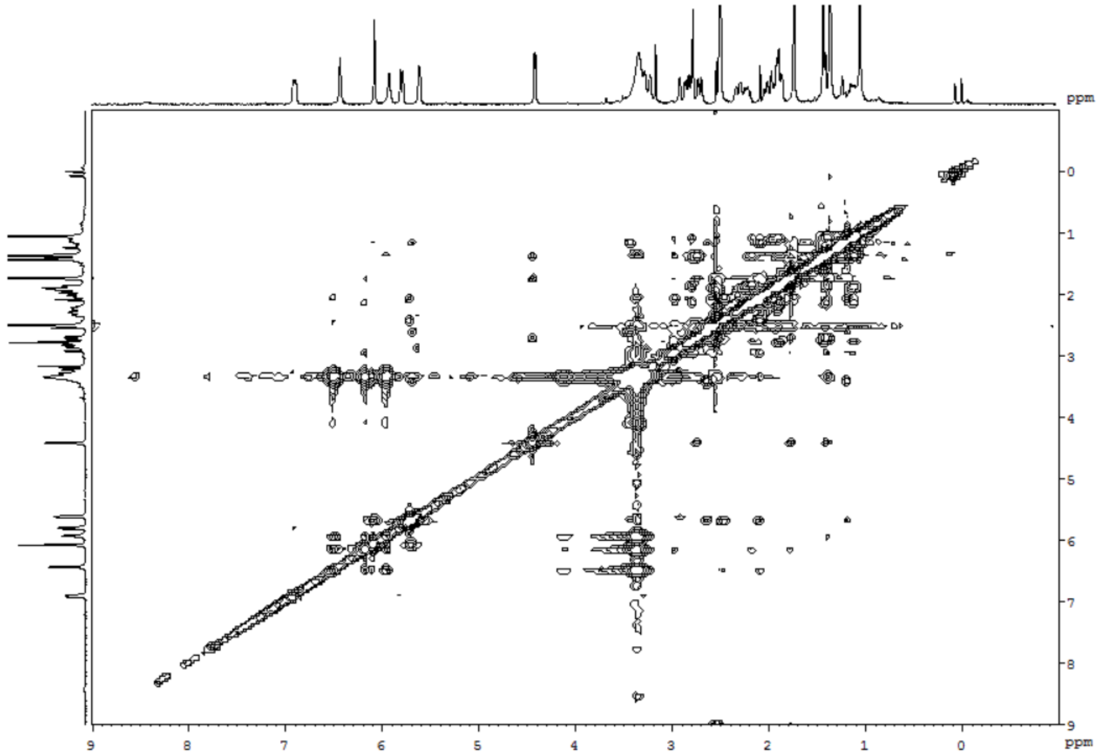

Supplementary Figure S15. NOESY spectrum of **2** (600 MHz, DMSO-d_6)

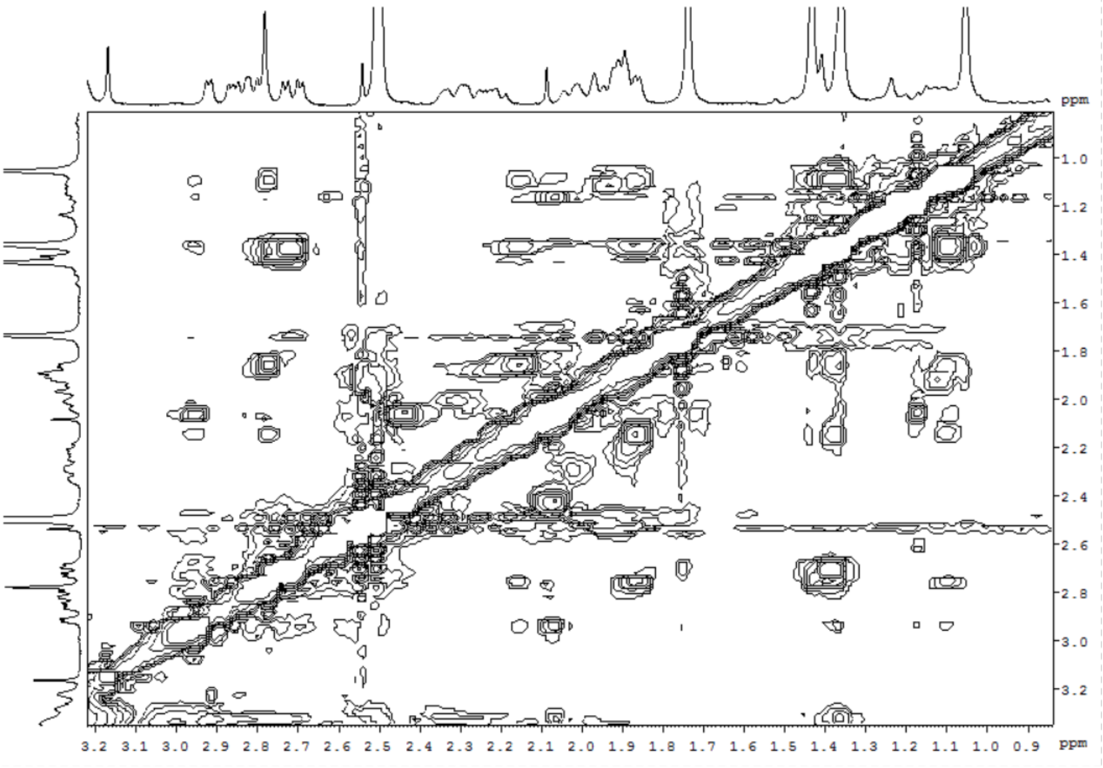

Supplementary Figure S16. Selected NOESY spectrum of **2** (600 MHz, DMSO-d_6)

Tolerance = 2.1 PPM / DBE: min = 3.0, max = 50.0
Element prediction: Off
Number of isotope peaks used for i-FIT = 3

Monoisotopic Mass, Even Electron Ions
250 formula(e) evaluated with 1 results within limits (up to 50 closest results for each mass)
Elements Used:
C: 0-100 H: 0-100 O: 0-200 Na: 0-1
SCPs12 neg 49 (0.205)
1: TOF MS ES-

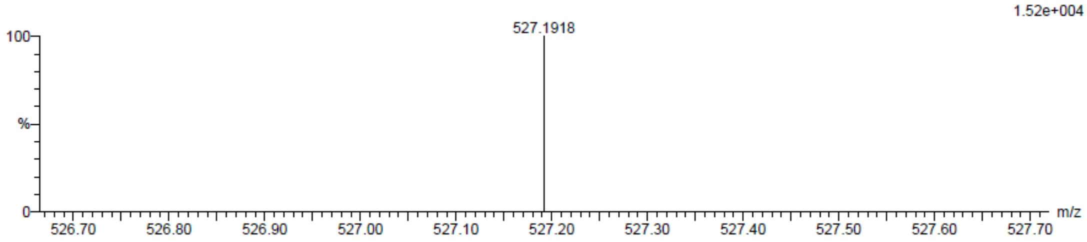

Minimum:				3.0					
Maximum:		4.0	2.1	50.0					
Mass	Calc. Mass	mDa	PPM	DBE	i-FIT	Norm	Conf(%)	Formula
527.1918	527.1917	0.1	0.2	13.5	31.5	n/a	n/a	C28 H31 O10

Supplementary Figure S17. HRESIMS spectrum of **2**

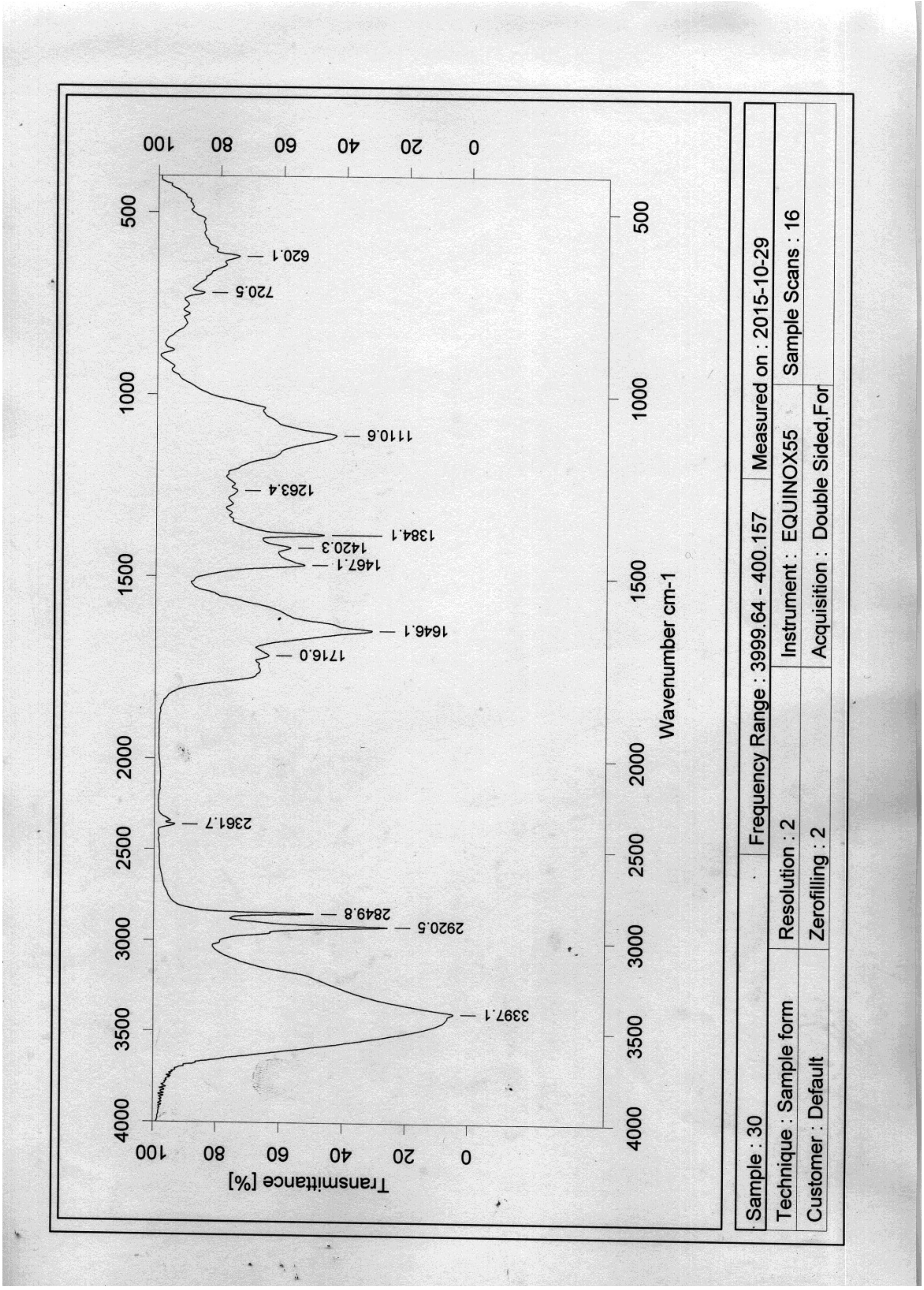

Supplementary Figure S18. IR spectrum of **2**

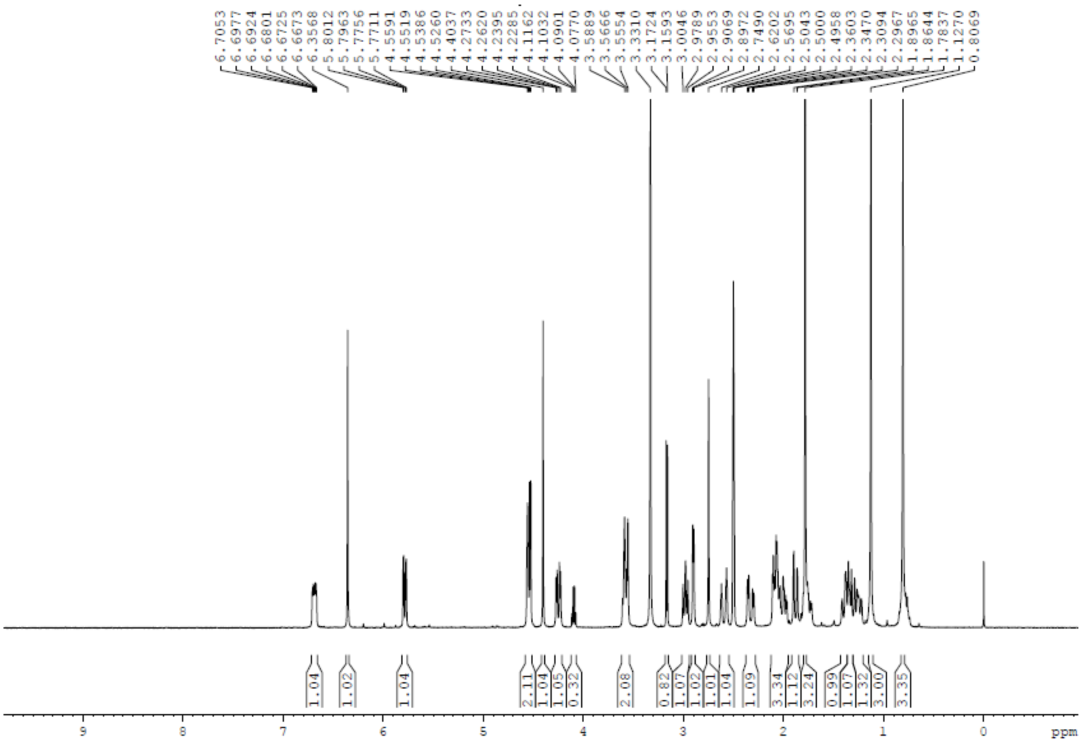

Supplementary Figure S19. ^{1}H NMR spectrum of **3** (400 MHz, DMSO-d_6)

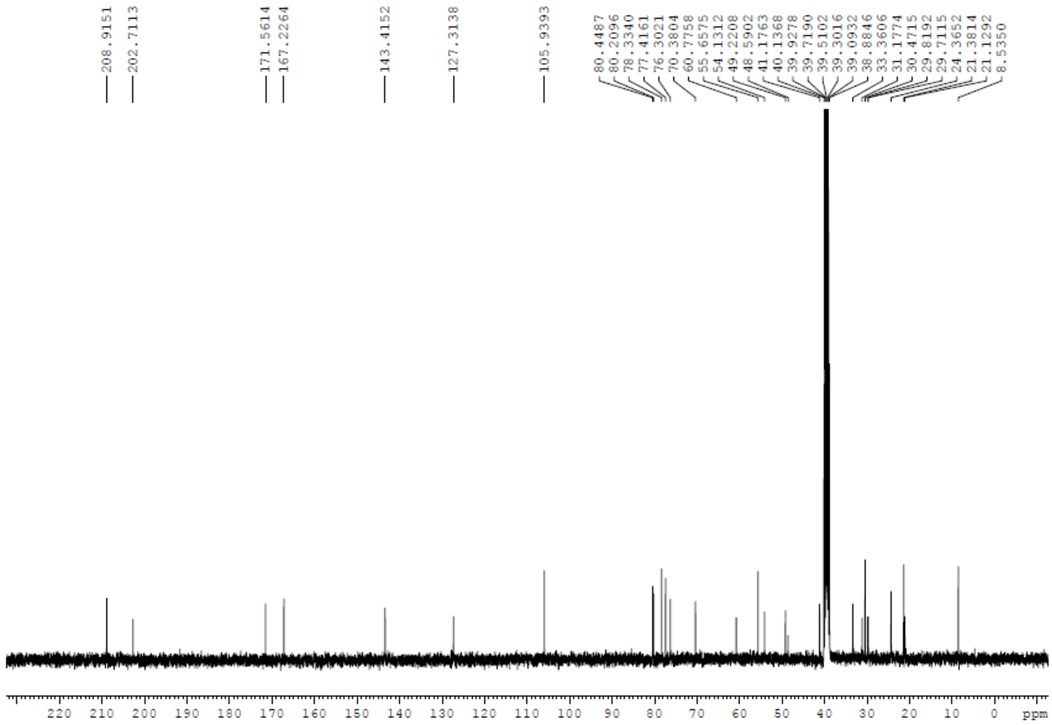

Supplementary Figure S20. ^{13}C NMR spectrum of **3** (100 MHz, DMSO-d_6)

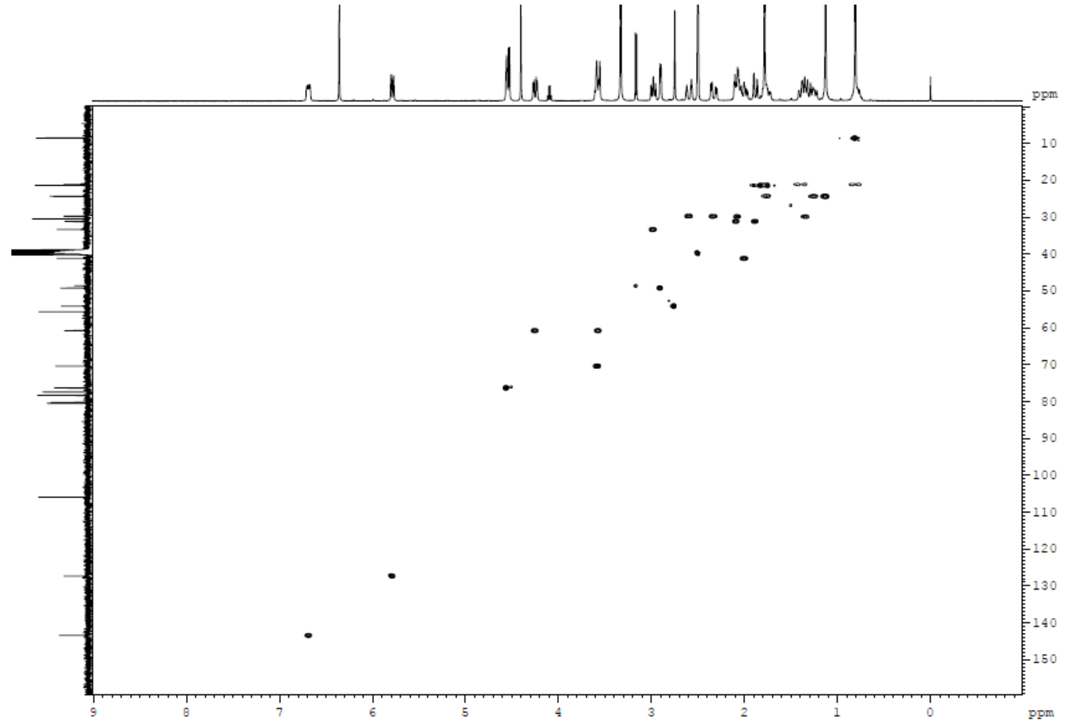

Supplementary Figure S21. HSQC spectrum of **3** (600 MHz, DMSO-d_6)

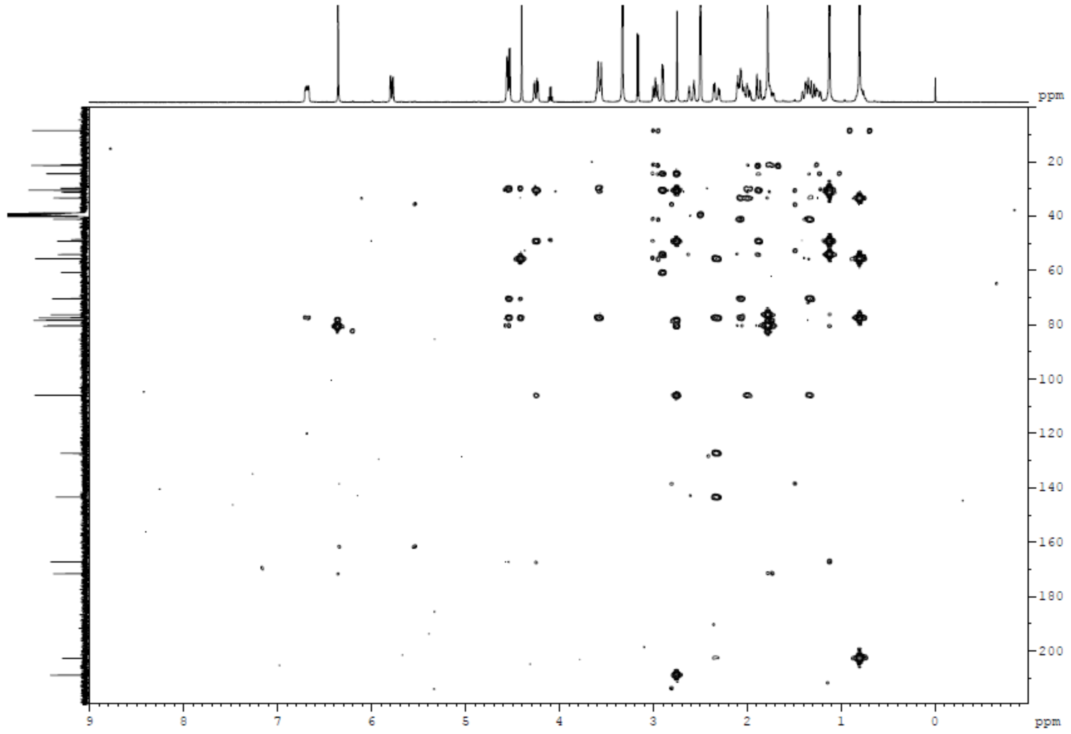

Supplementary Figure S22. HMBC spectrum of **3** (600 MHz, DMSO-d_6)

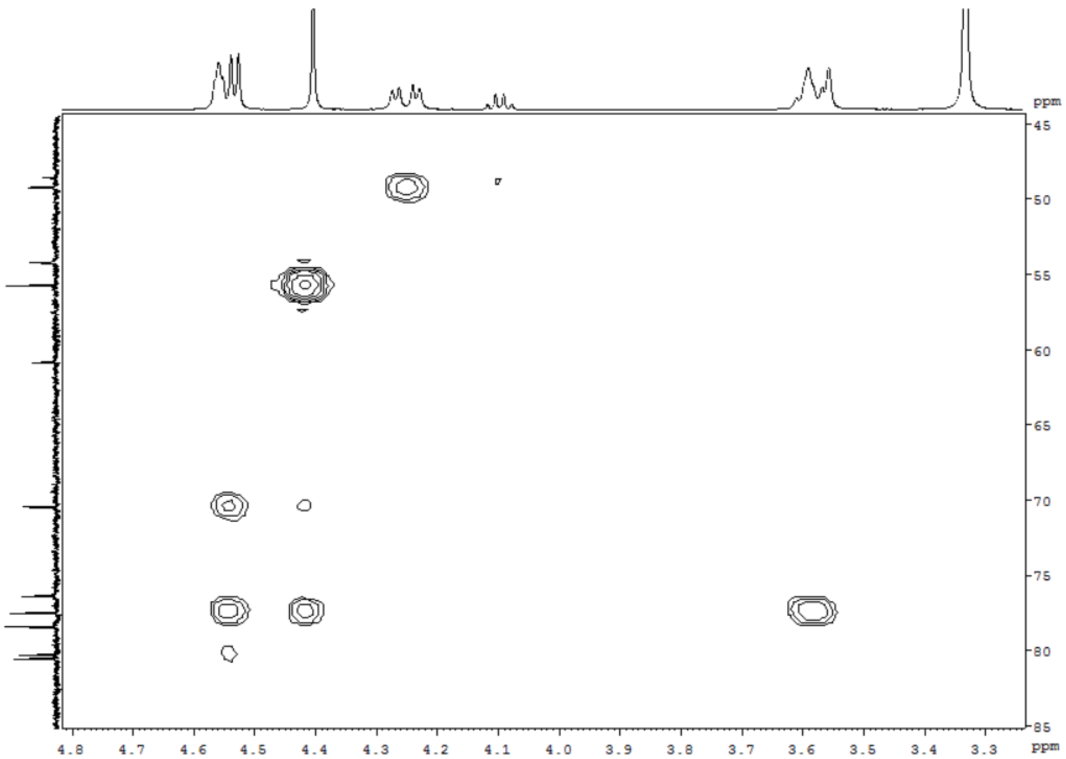

Supplementary Figure S23. Selected HMBC spectrum of **3** (600 MHz, DMSO-d_6)

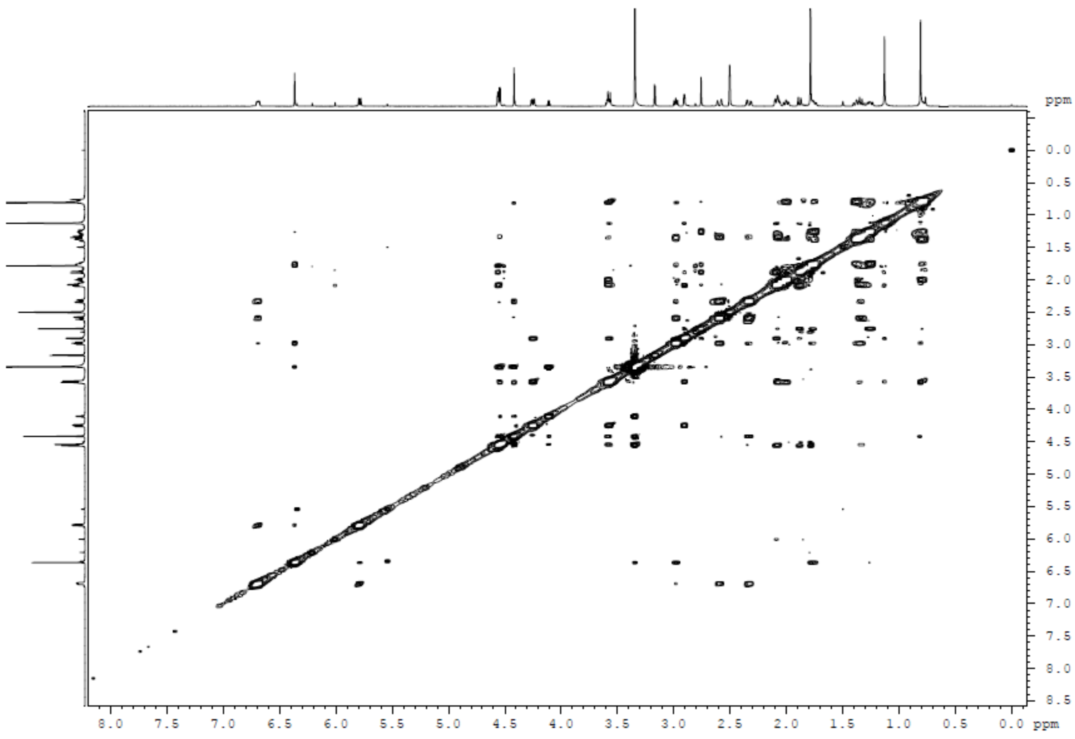

Supplementary Figure S24. NOESY spectrum of **3** (600 MHz, DMSO-d_6)

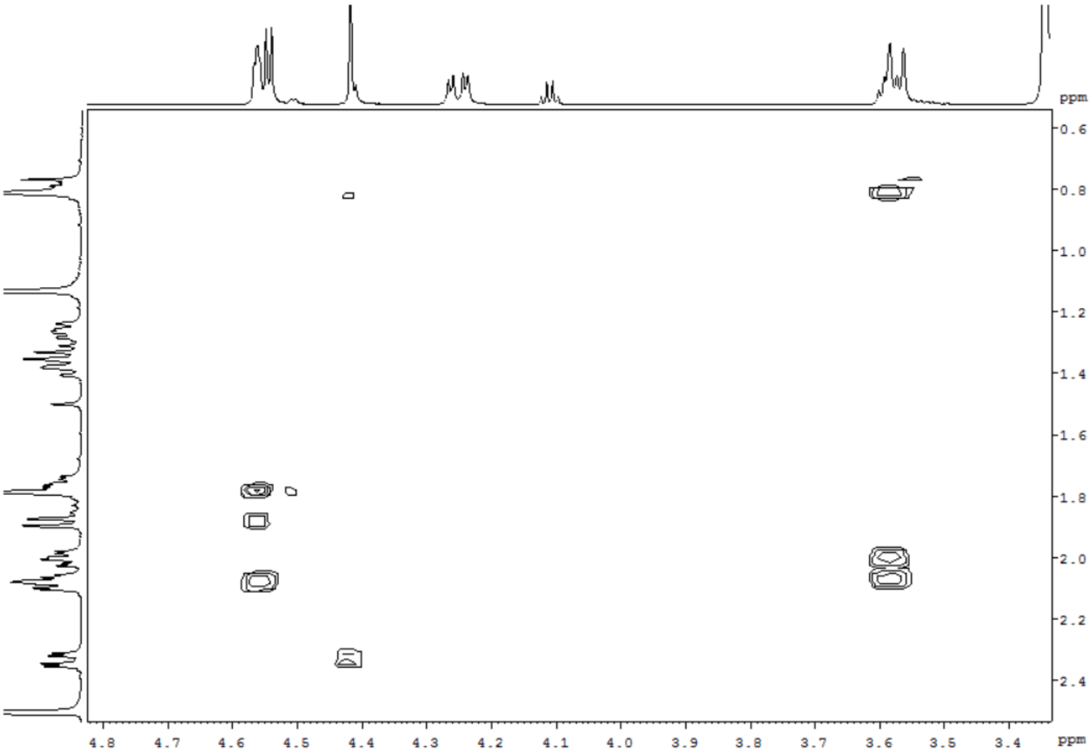

Supplementary Figure S25. Selected NOESY spectrum of **3** (600 MHz, DMSO-d_6)

Tolerance = 2.0 PPM / DBE: min = 3.0, max = 50.0
Element prediction: Off
Number of isotope peaks used for i-FIT = 3

Monoisotopic Mass, Even Electron Ions
139 formula(e) evaluated with 1 results within limits (up to 50 closest results for each mass)
Elements Used:
C: 0-100 H: 0-100 O: 0-200
SCPs23 neg 41 (0.170) Cm (41:55)
1: TOF MS ES-

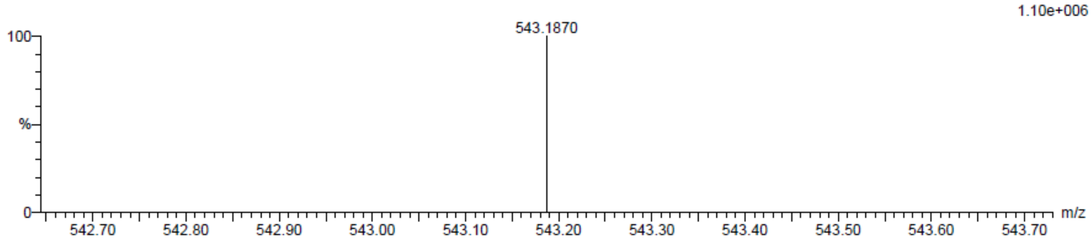

Minimum:				3.0					
Maximum:		4.0	2.0	50.0					
Mass	Calc. Mass	mDa	PPM	DBE	i-FIT	Norm	Conf(%)	Formula
543.1870	543.1866	0.4	0.7	13.5	42.4	n/a	n/a	C28 H31 O11

Supplementary Figure S26. HRESIMS spectrum of **3**

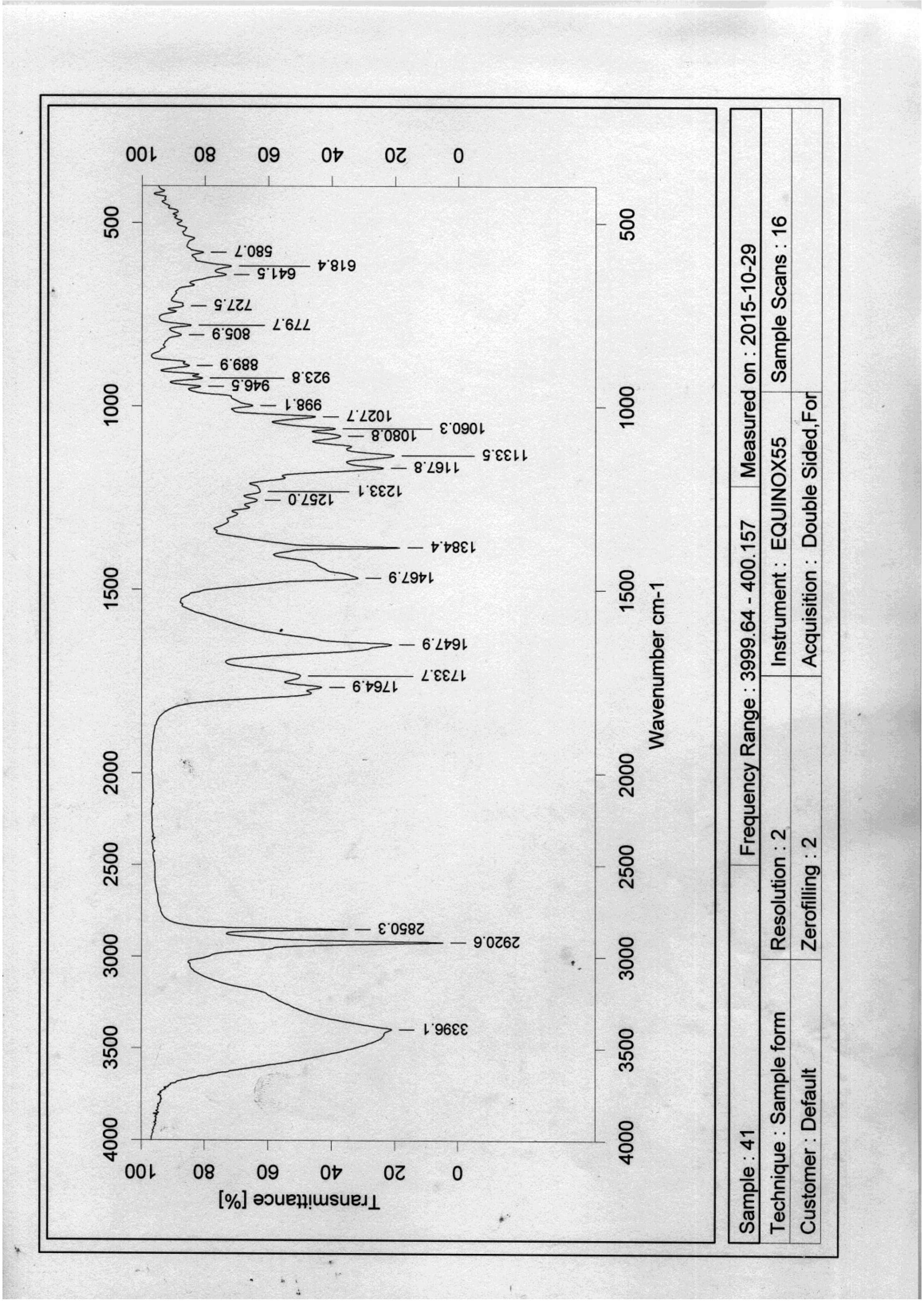

Supplementary Figure S27. IR spectrum of **3**

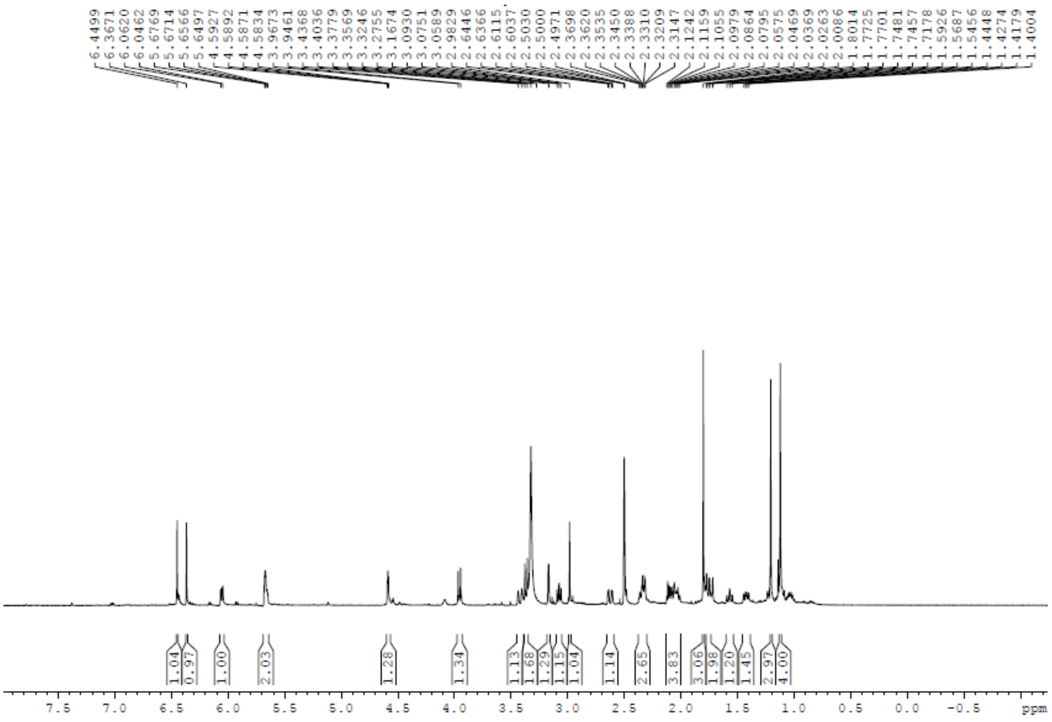

Supplementary Figure S28. ^{1}H NMR spectrum of **4** (600 MHz, DMSO-d_6)

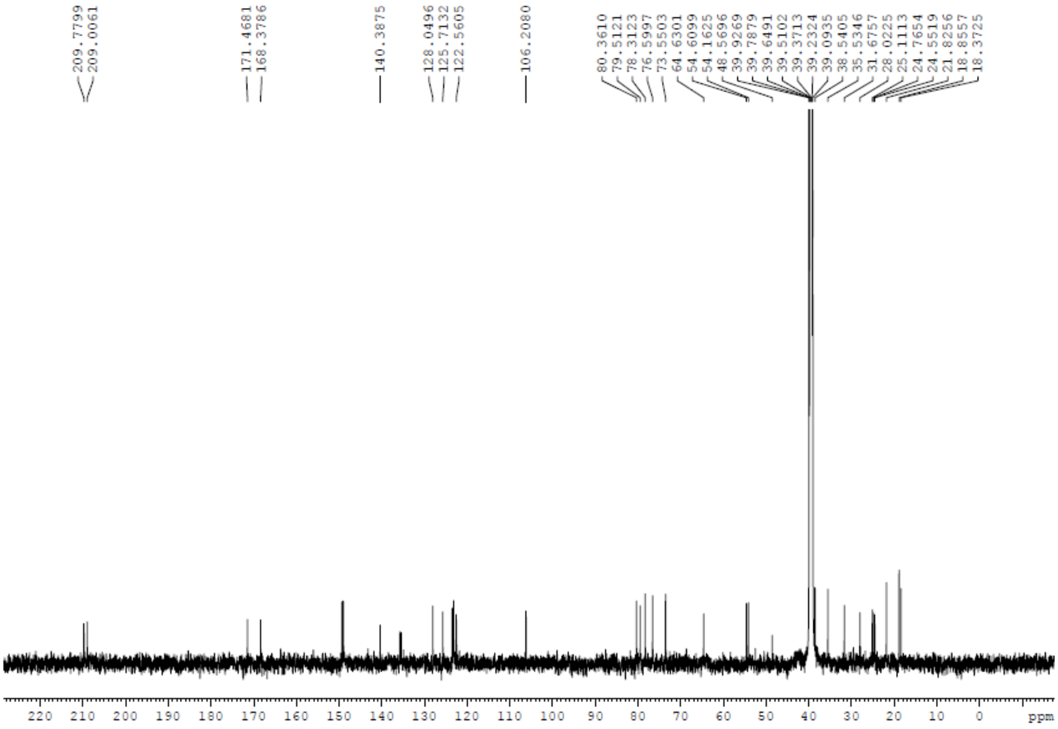

Supplementary Figure S29. ^{13}C NMR spectrum of **4** (150 MHz, DMSO-d_6)

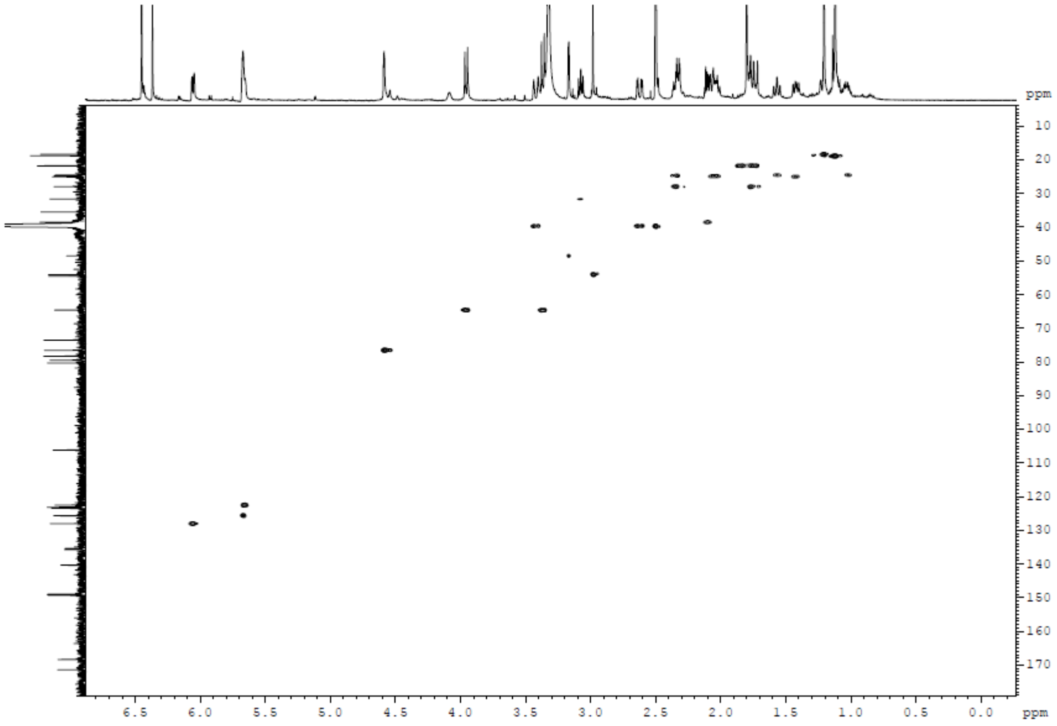

Supplementary Figure S30. HSQC spectrum of **4** (600 MHz, DMSO-d_6)

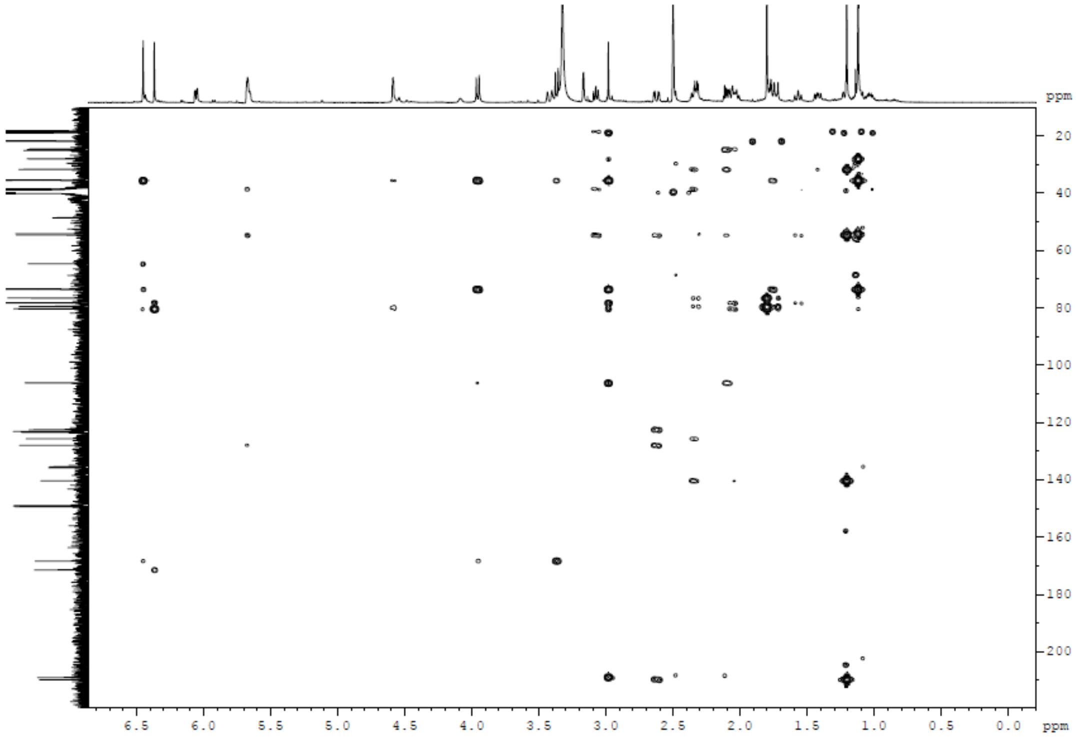

Supplementary Figure S31. HMBC spectrum of **4** (600 MHz, DMSO-d_6)

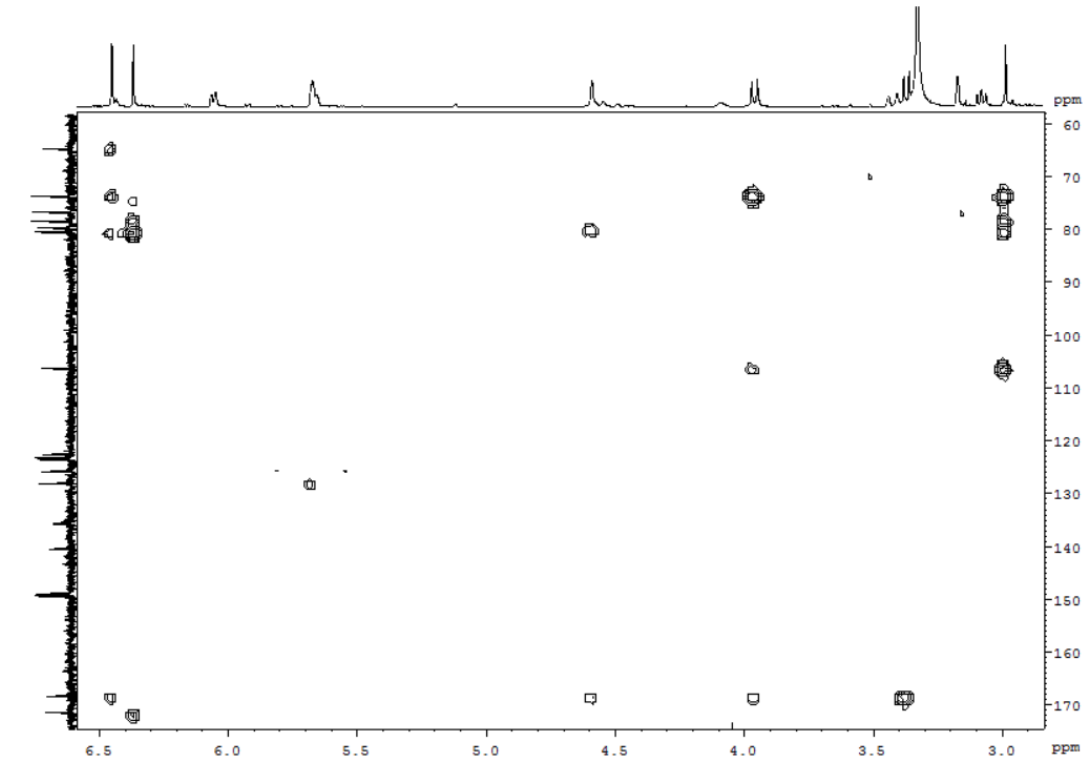

Supplementary Figure S32. Selected HMBC spectrum of **4** (600 MHz, DMSO-d_6)

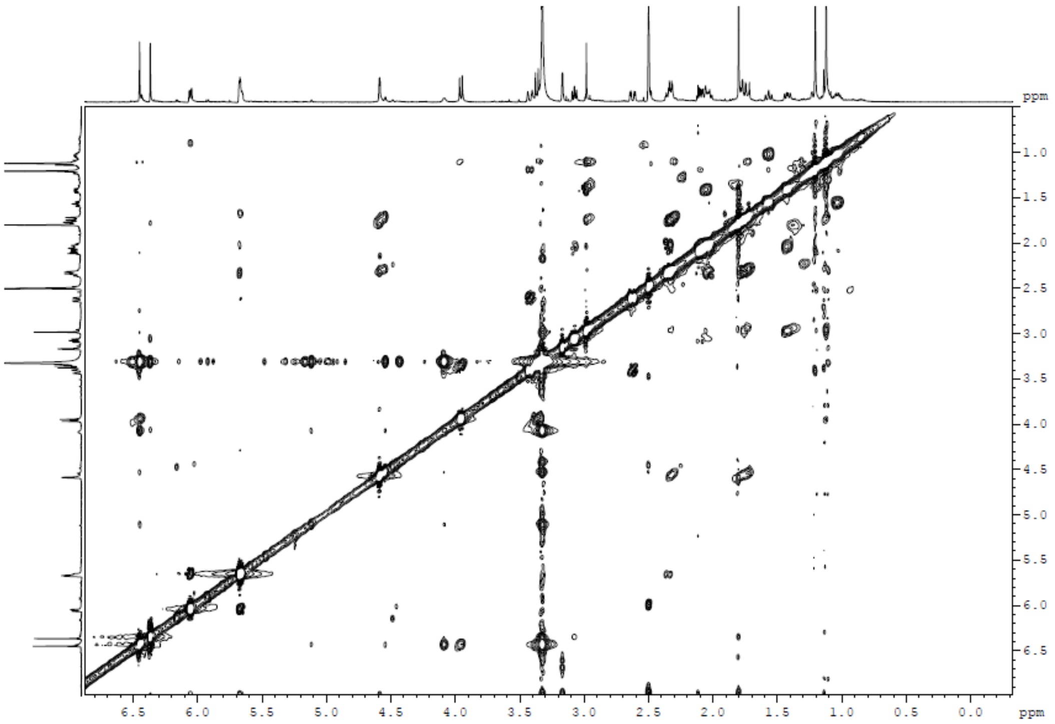

Supplementary Figure S33. NOESY spectrum of **4** (600 MHz, DMSO-d_6)

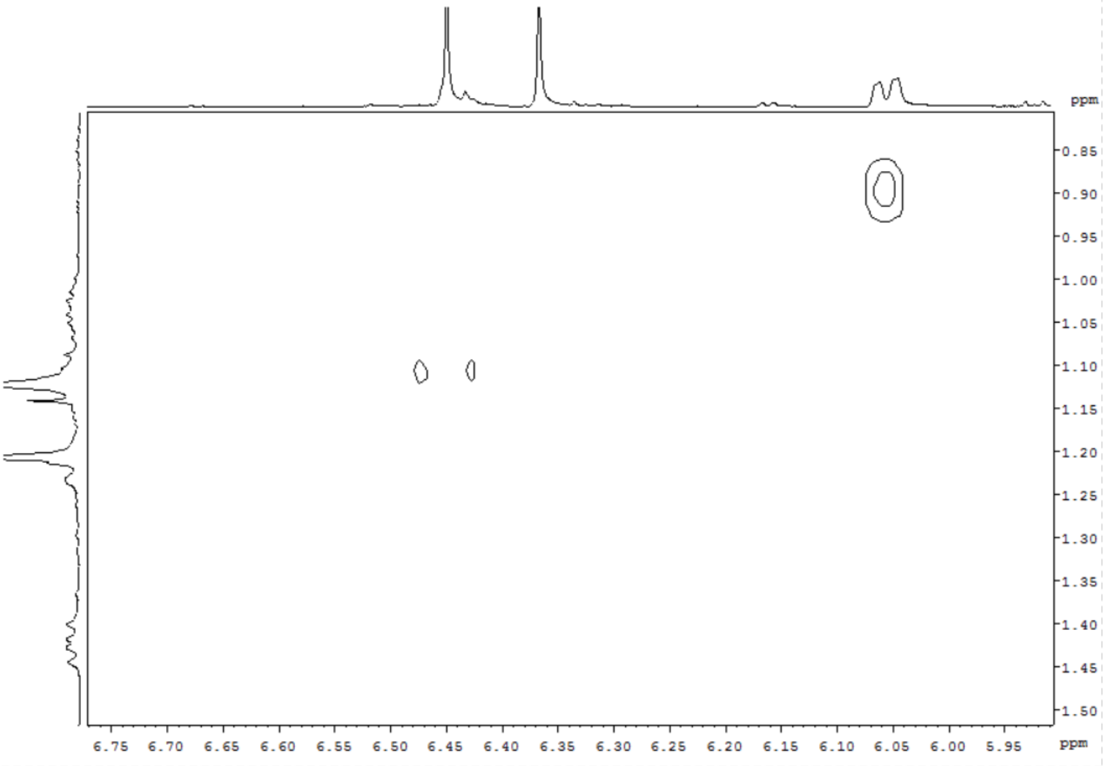

Supplementary Figure S34. Selected NOESY spectrum of **4** (600 MHz, DMSO-d_6)

Tolerance = 2.1 PPM / DBE: min = 3.0, max = 50.0
Element prediction: Off
Number of isotope peaks used for i-FIT = 3

Monoisotopic Mass, Even Electron Ions
238 formula(e) evaluated with 1 results within limits (up to 50 closest results for each mass)
Elements Used:
C: 0-100 H: 0-100 O: 0-200 Na: 0-1
SCPs13 neg 24 (0.109)
1: TOF MS ES-

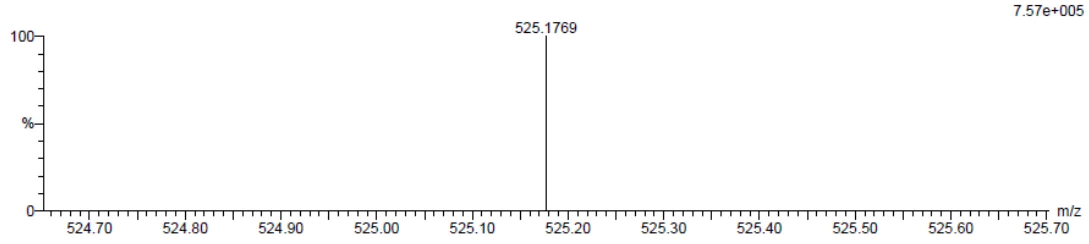

Minimum:				3.0				
Maximum:		4.0	2.1	50.0				
Mass	Calc. Mass	mDa	PPM	DBE	i-FIT	Norm	Conf(%)	Formula
525.1769	525.1761	0.8	1.5	14.5	41.9	n/a	n/a	C28 H29 O10

Supplementary Figure S35. HRESIMS spectrum of **4**

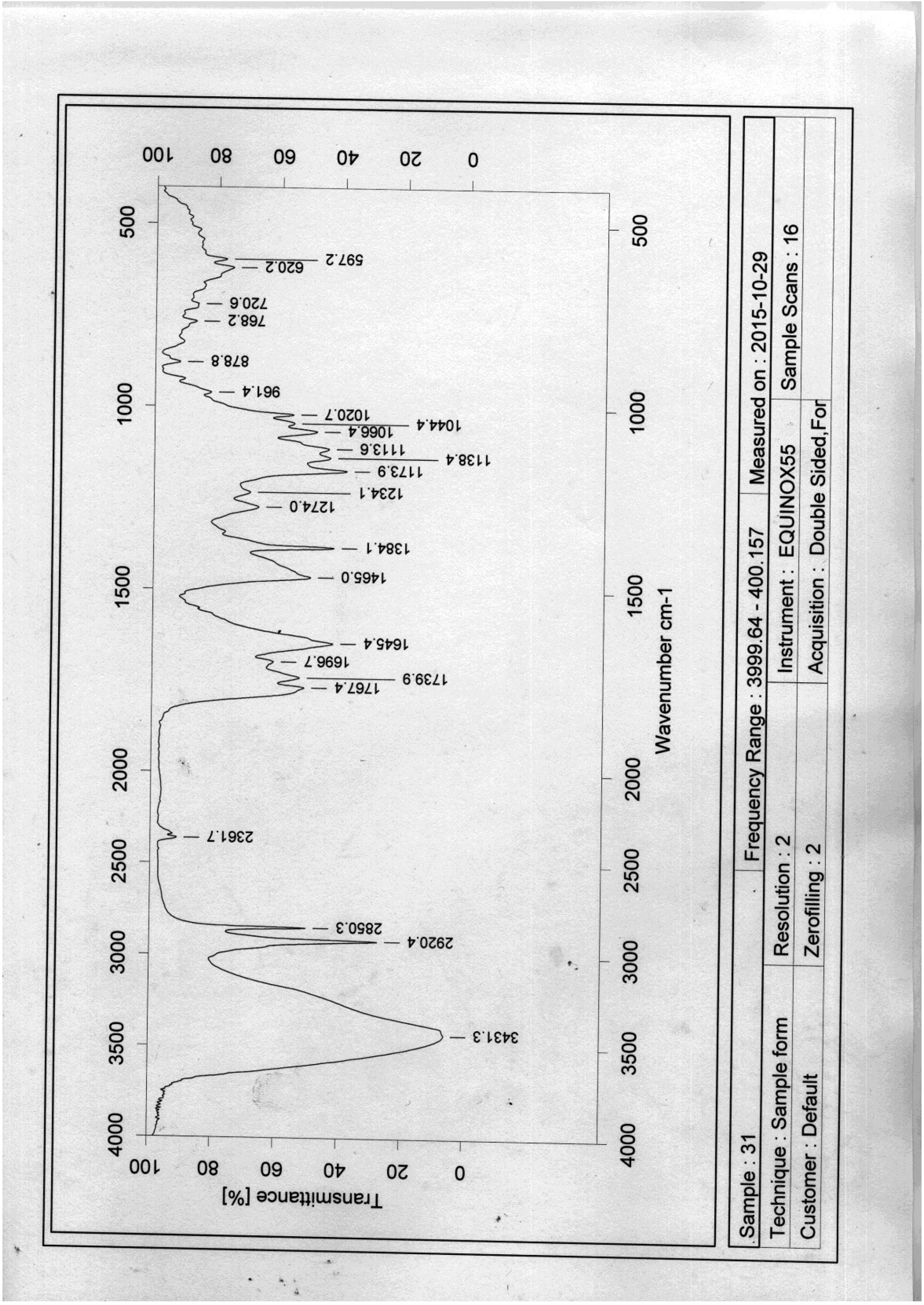

Supplementary Figure S36. IR spectrum of **4**

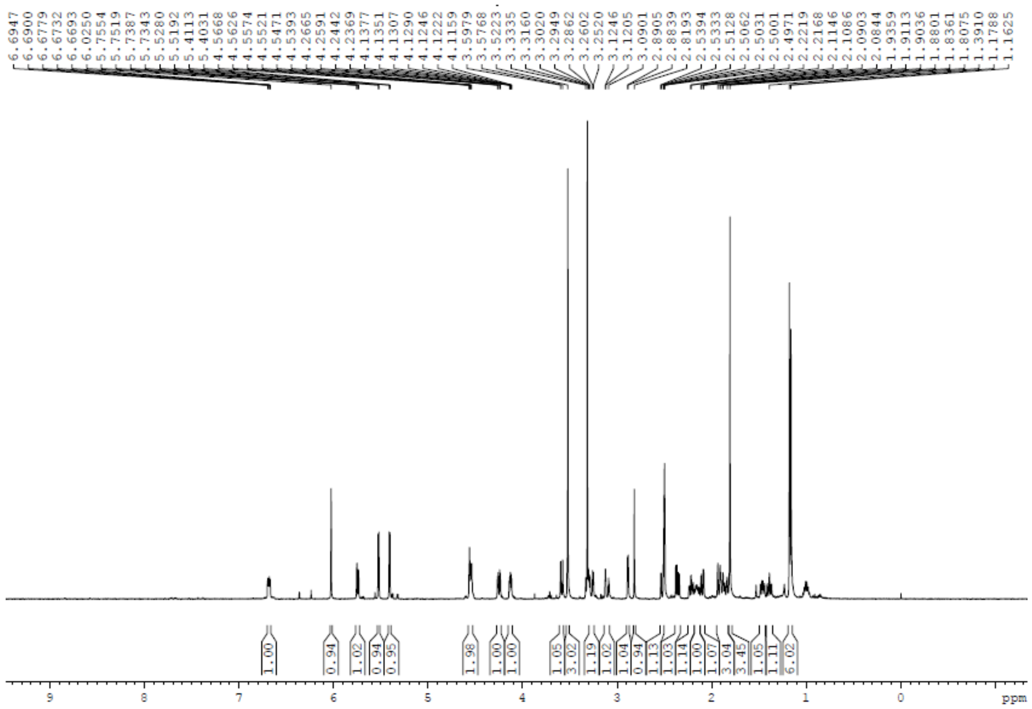

Supplementary Figure S37. ^{1}H NMR spectrum of **5** (400 MHz, DMSO-d_6)

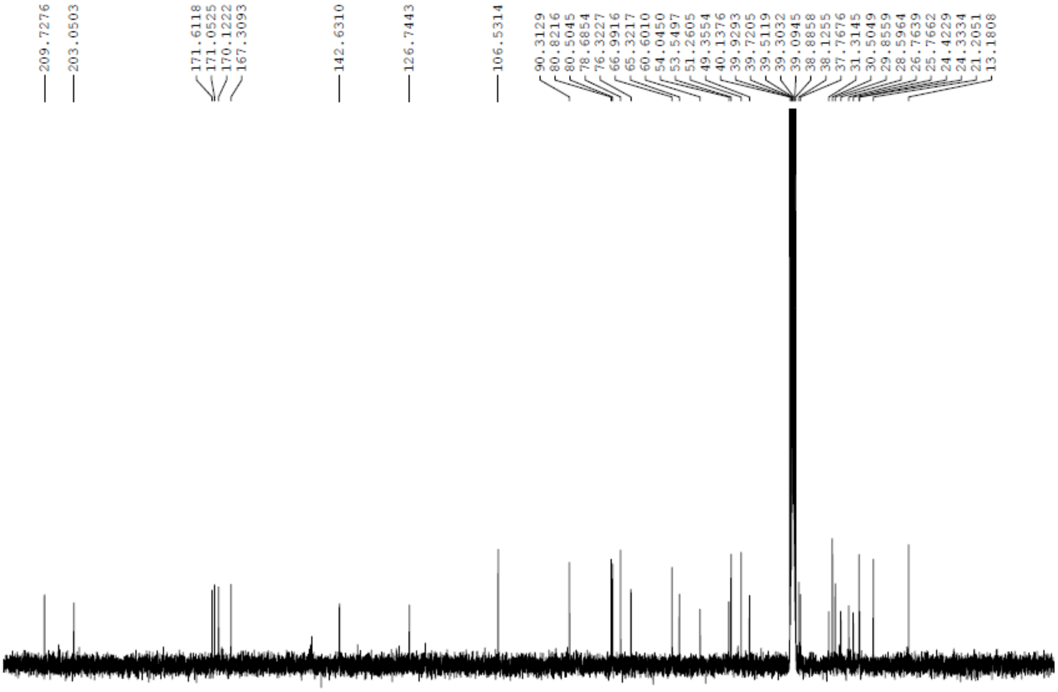

Supplementary Figure S38. ^{13}C NMR spectrum of **5** (100 MHz, DMSO-d_6)

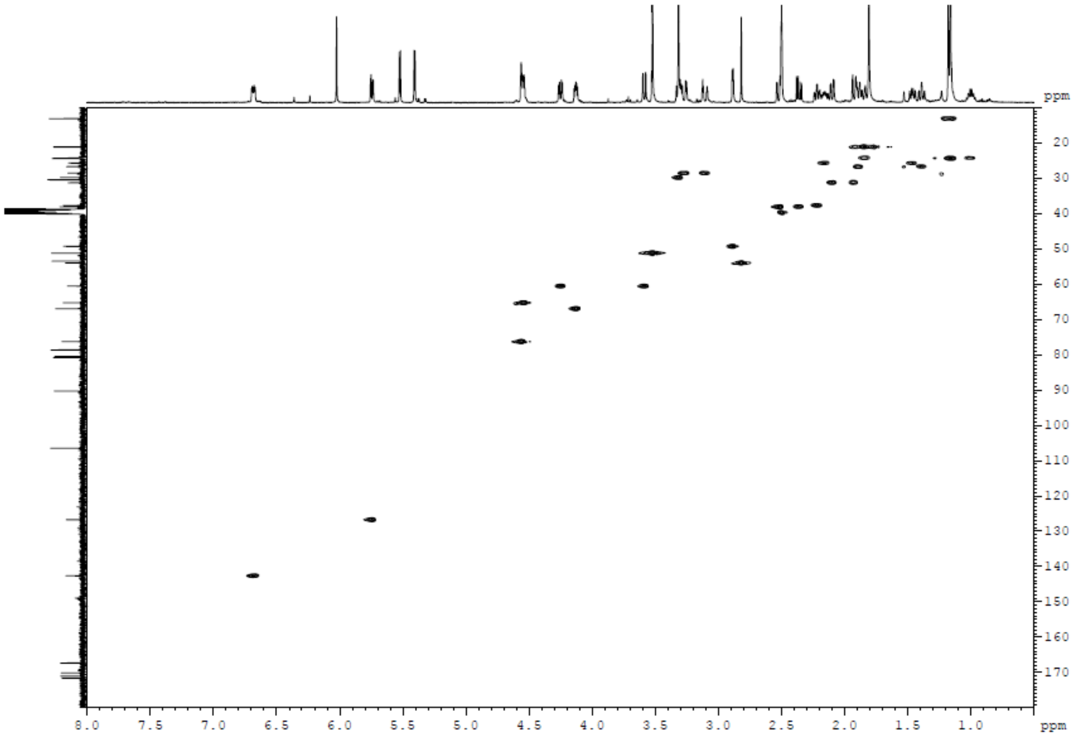

Supplementary Figure S39. HSQC spectrum of **5** (600 MHz, DMSO-d_6)

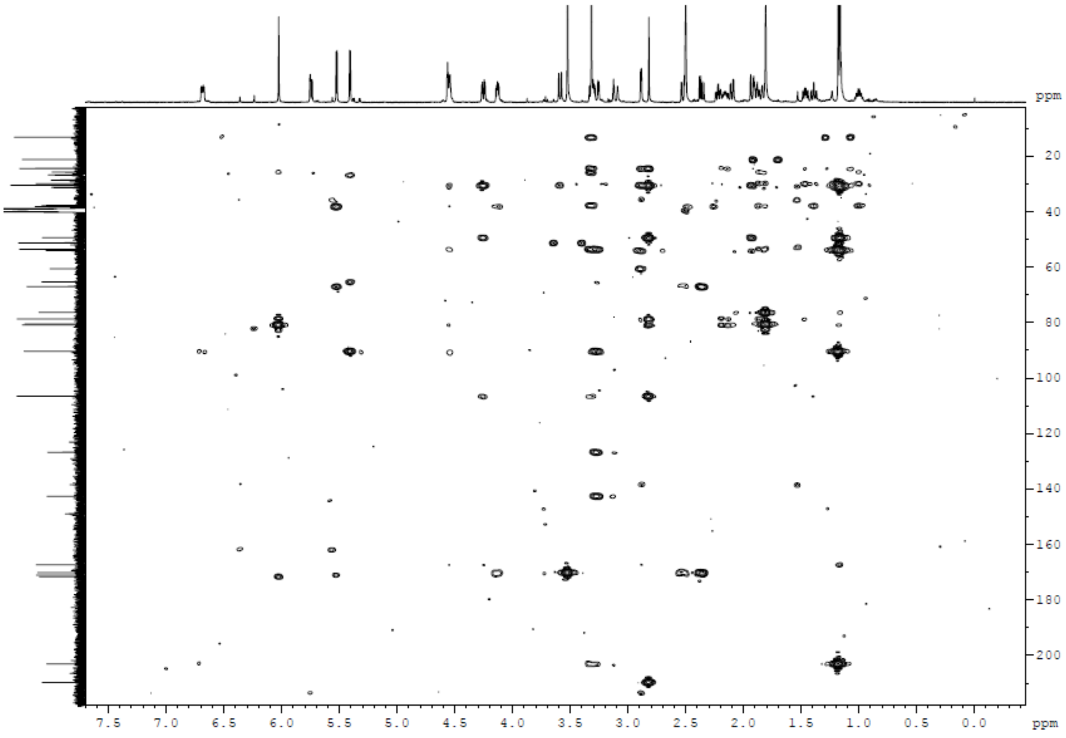

Supplementary Figure S40. HMBC spectrum of **5** (600 MHz, DMSO-d_6)

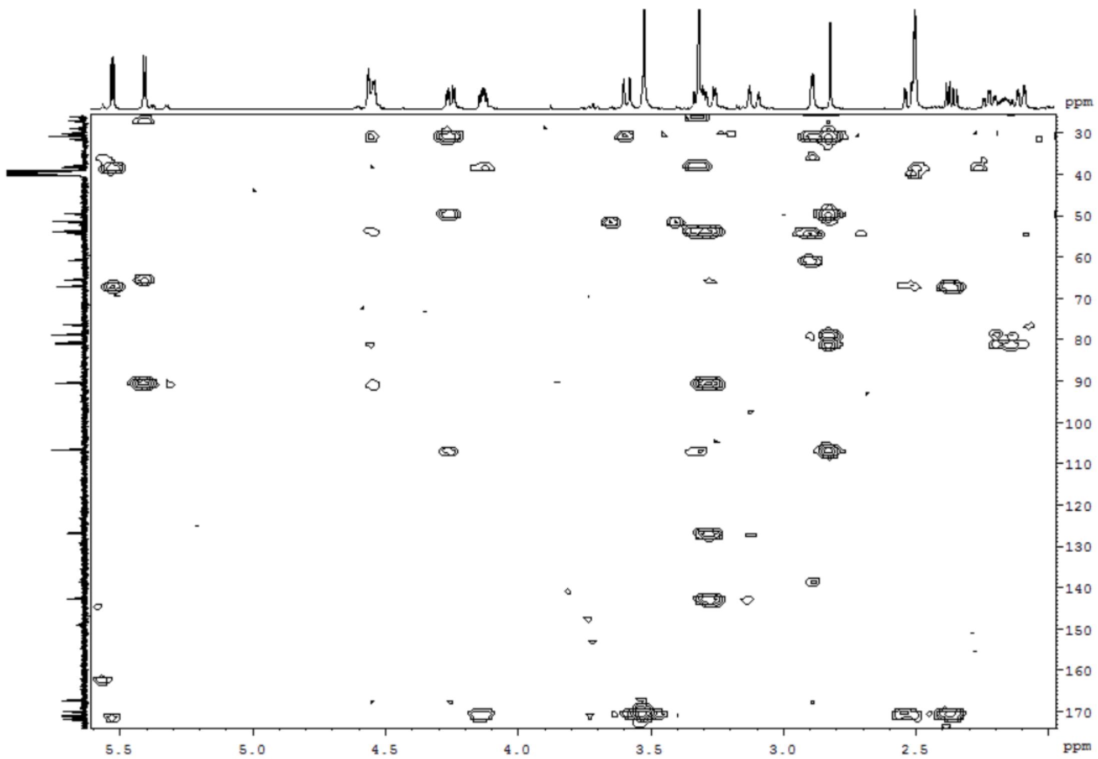

Supplementary Figure S41. Selected HMBC spectrum of **5** (600 MHz, DMSO-d_6)

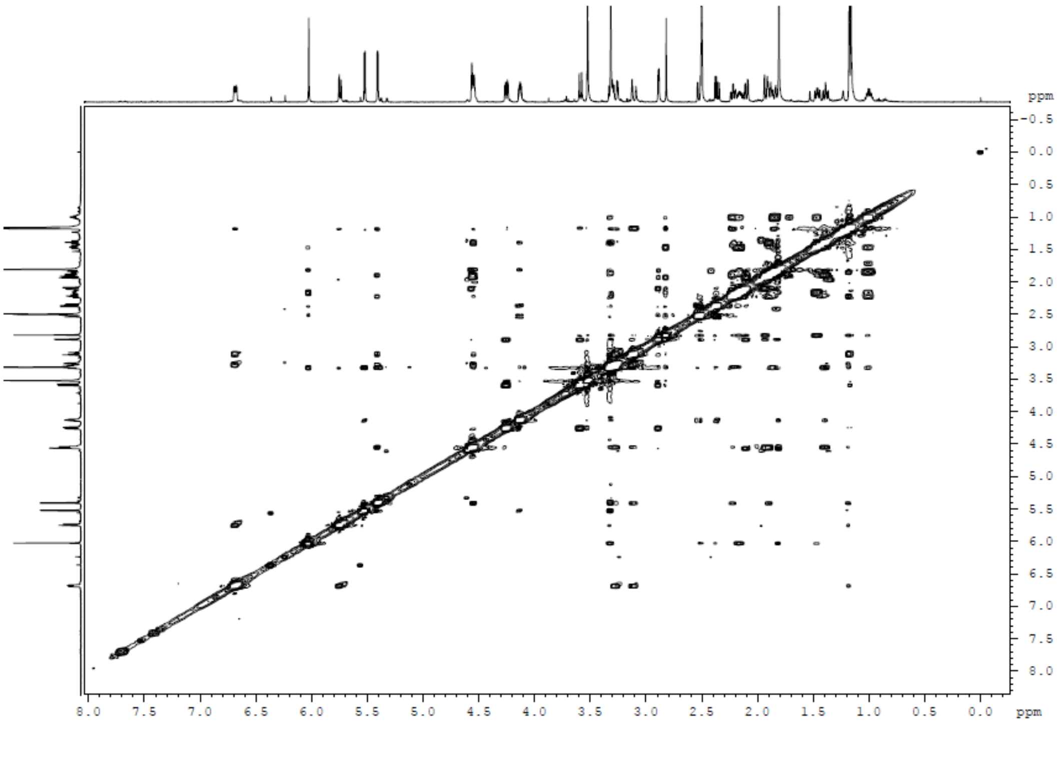

Supplementary Figure S42. NOESY spectrum of **5** (600 MHz, DMSO-d_6)

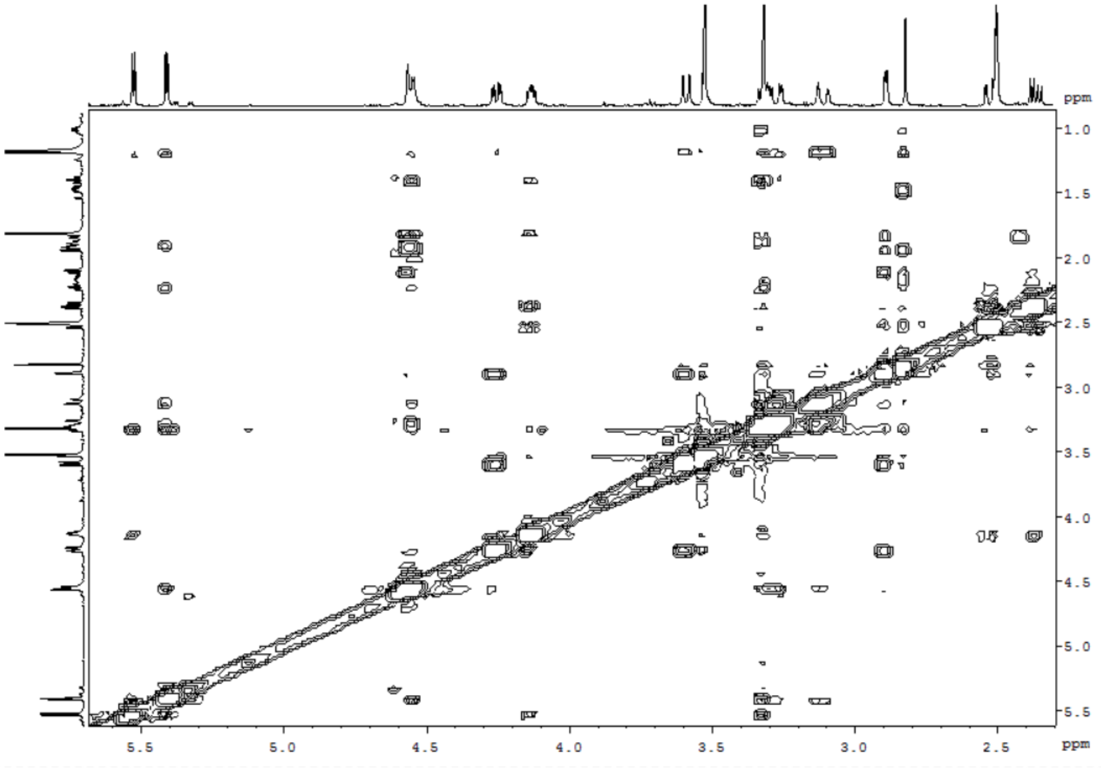

Supplementary Figure S43. Selected NOESY spectrum of **5** (600 MHz, DMSO-d_6)

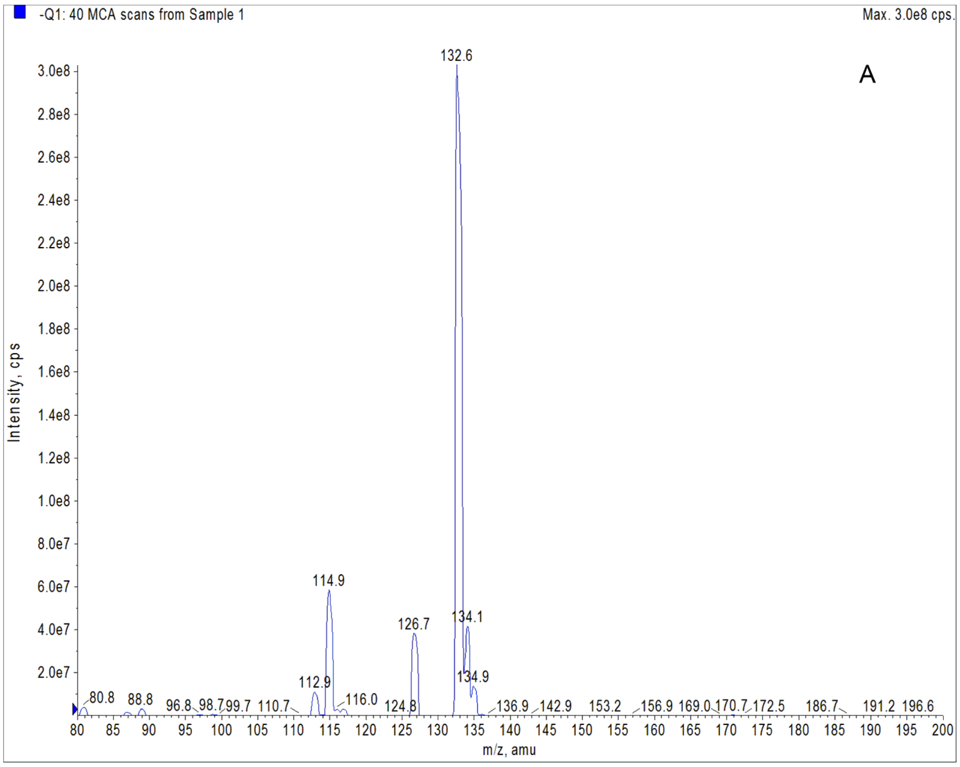

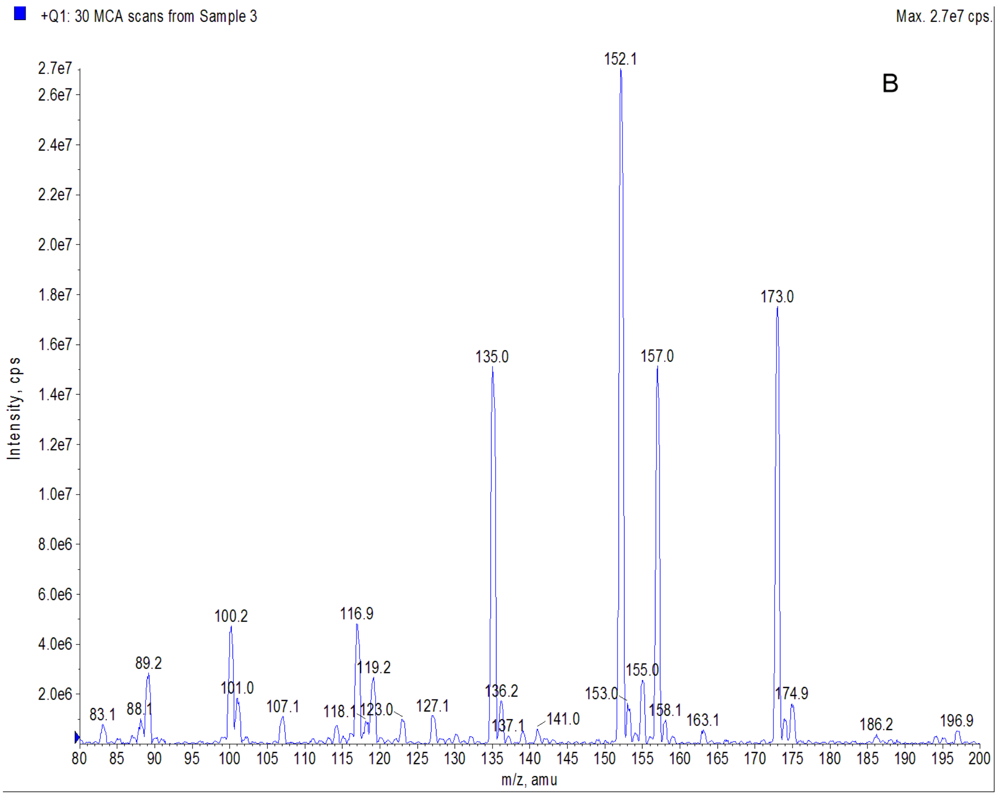

Supplementary Figure S44. MS spectrum (A, negative mode; B, positive mode) of the hydrolyzed product for **5**

Tolerance = 2.1 PPM / DBE: min = 3.0, max = 50.0
Element prediction: Off
Number of isotope peaks used for i-FIT = 3

Monoisotopic Mass, Even Electron Ions
380 formula(e) evaluated with 1 results within limits (up to 50 closest results for each mass)
Elements Used:
C: 0-100 H: 0-100 O: 0-200 Na: 0-1
SCPs18 neg 26 (0.116)
1: TOF MS ES-

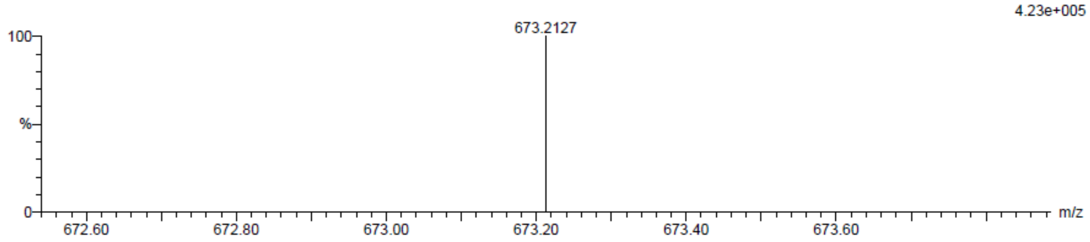

Minimum:				3.0				
Maximum:		4.0	2.1	50.0				
Mass	Calc. Mass	mDa	PPM	DBE	i-FIT	Norm	Conf(%)	Formula
673.2127	673.2132	-0.5	-0.7	15.5	40.4	n/a	n/a	C33 H37 O15

Supplementary Figure S45. HRESIMS spectrum of **5**

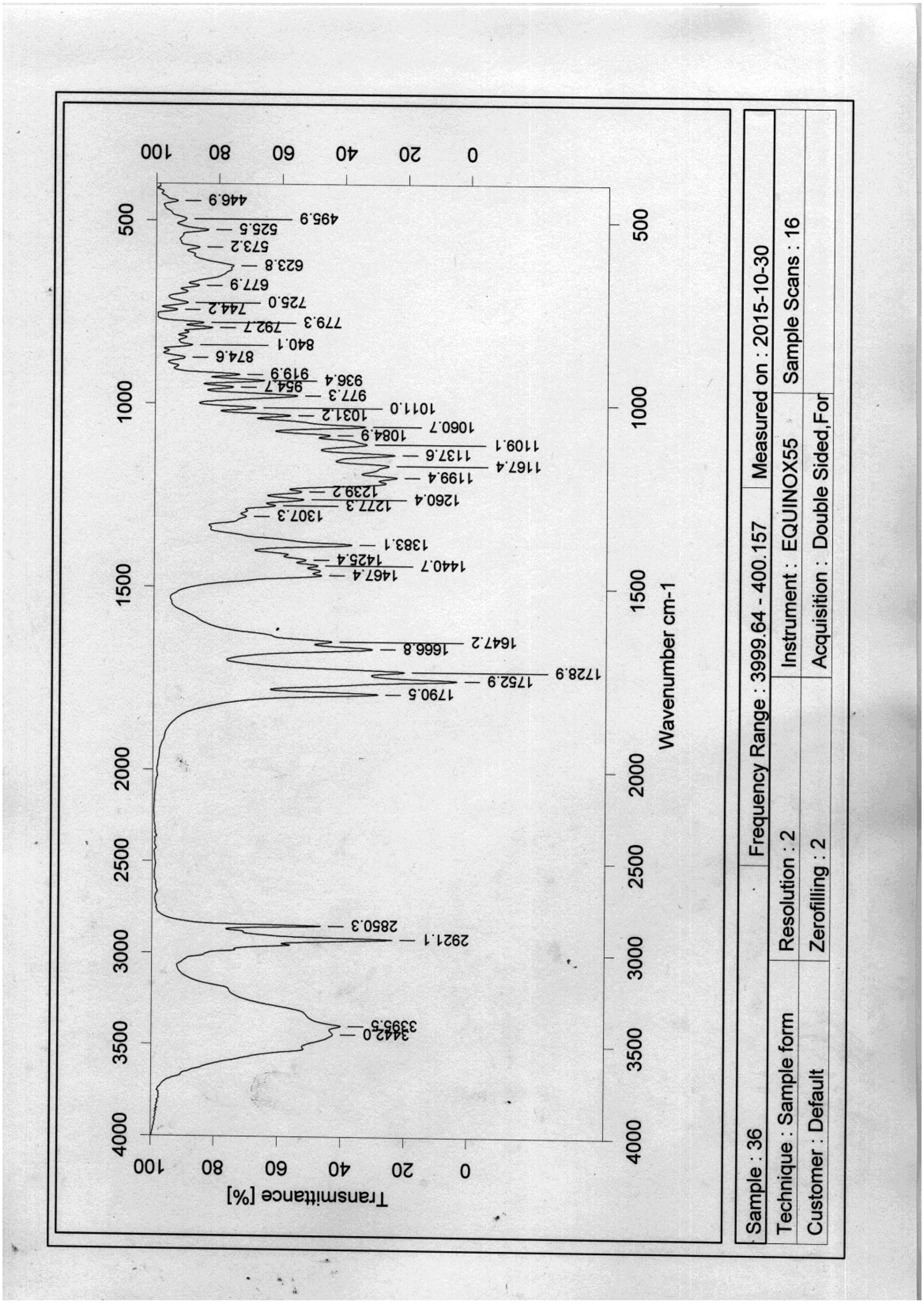

Supplementary Figure S46. IR spectrum of **5**

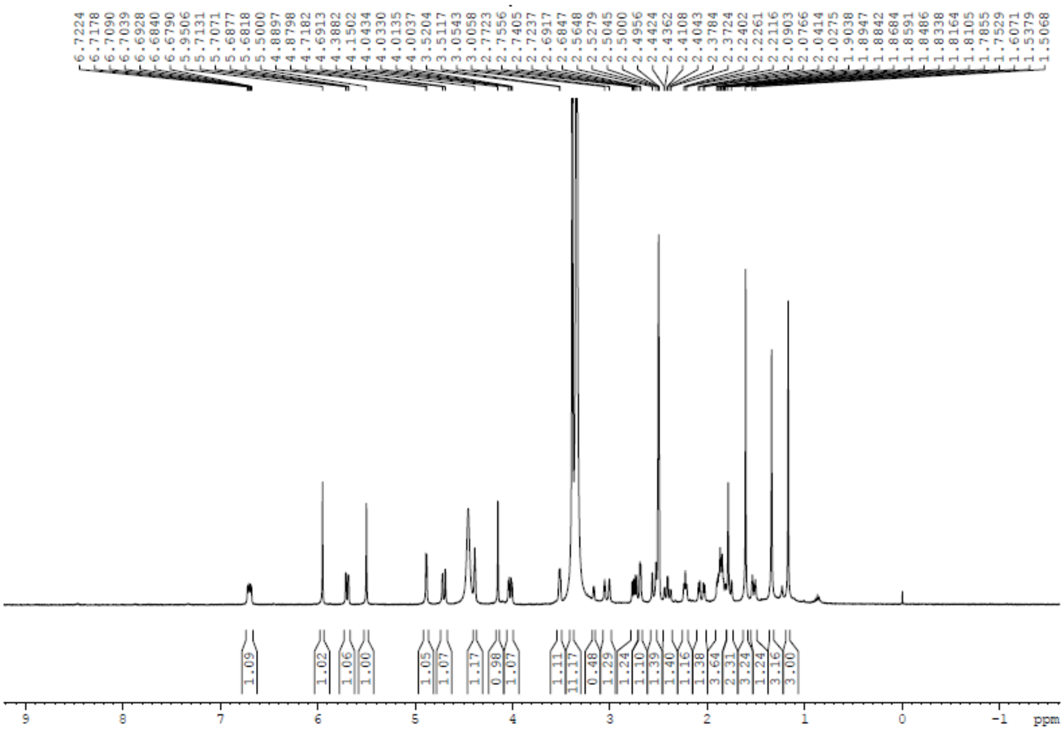

Supplementary Figure S47. ^{1}H NMR spectrum of **6** (600 MHz, DMSO-d_6)

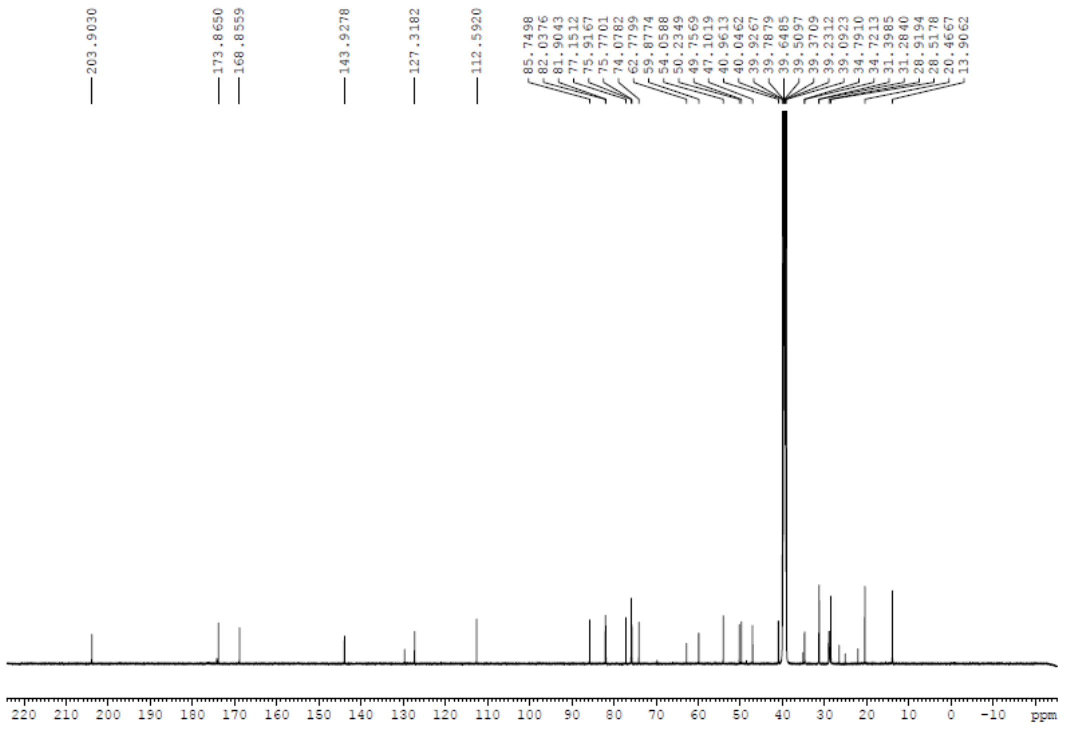

Supplementary Figure S48. ^{13}C NMR spectrum of **6** (150 MHz, DMSO-d_6)

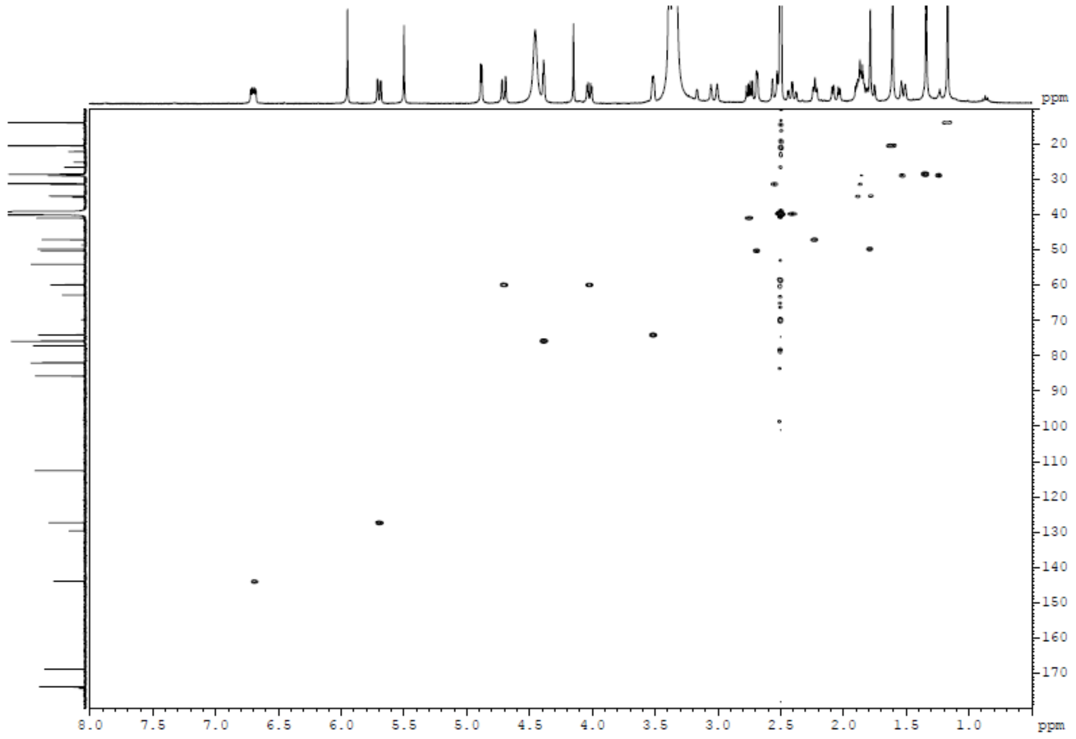

Supplementary Figure S49. HSQC spectrum of **6** (600 MHz, DMSO-d_6)

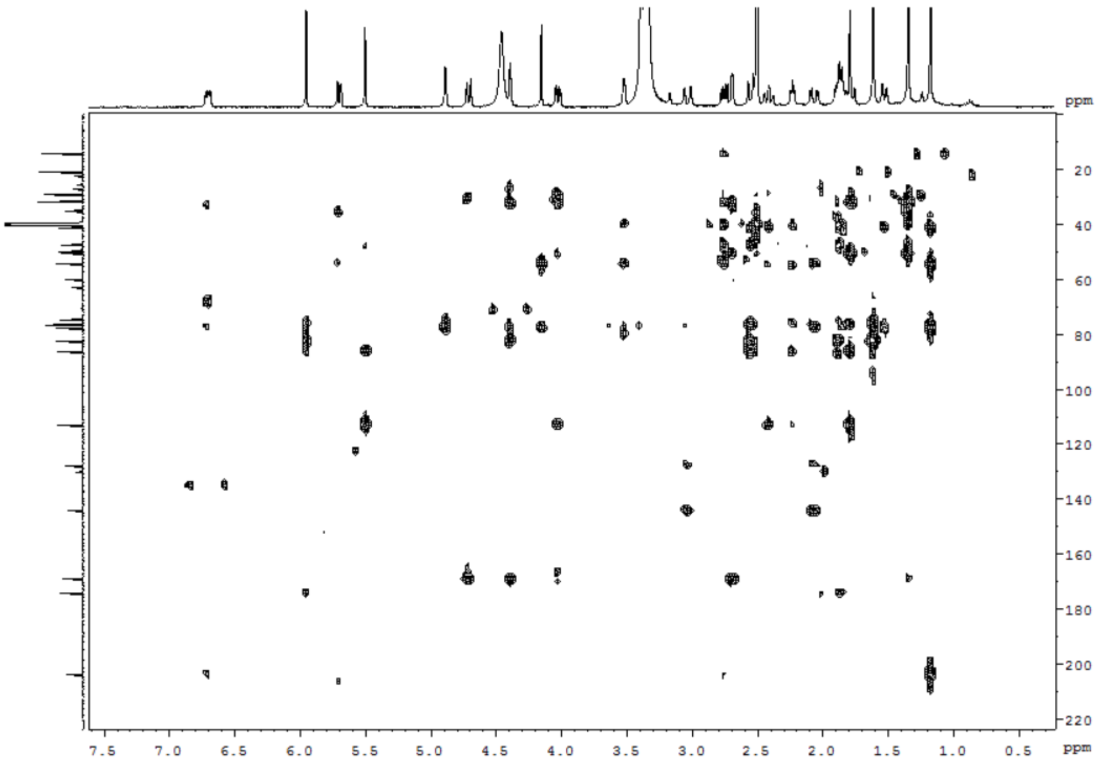

Supplementary Figure S50. HMBC spectrum of **6** (600 MHz, DMSO-d_6)

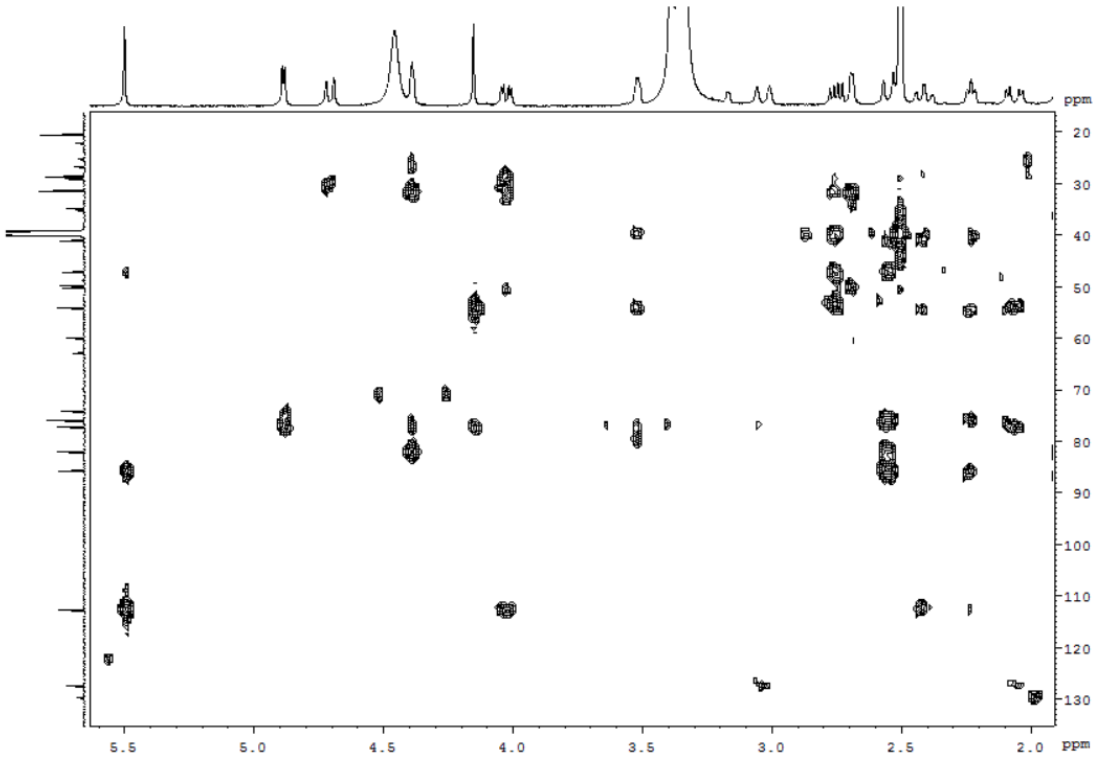

Supplementary Figure S51. Selected HMBC spectrum of **6** (600 MHz, DMSO-d_6)

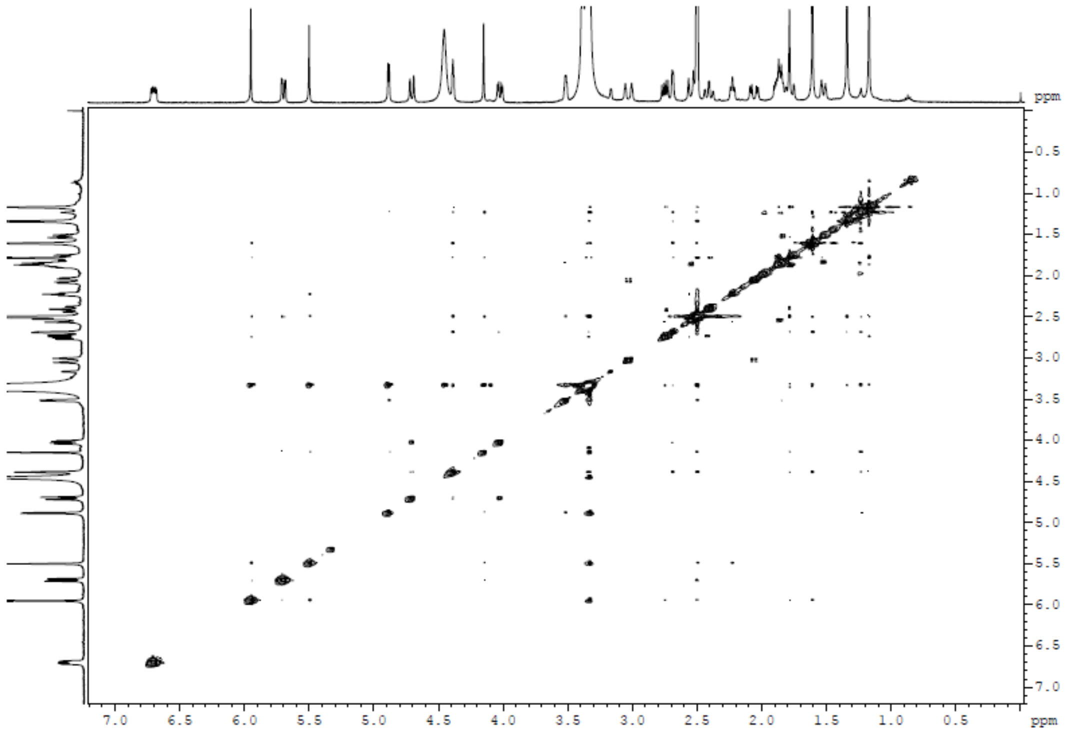

Supplementary Figure S52. NOESY spectrum of **6** (600 MHz, DMSO-d_6)

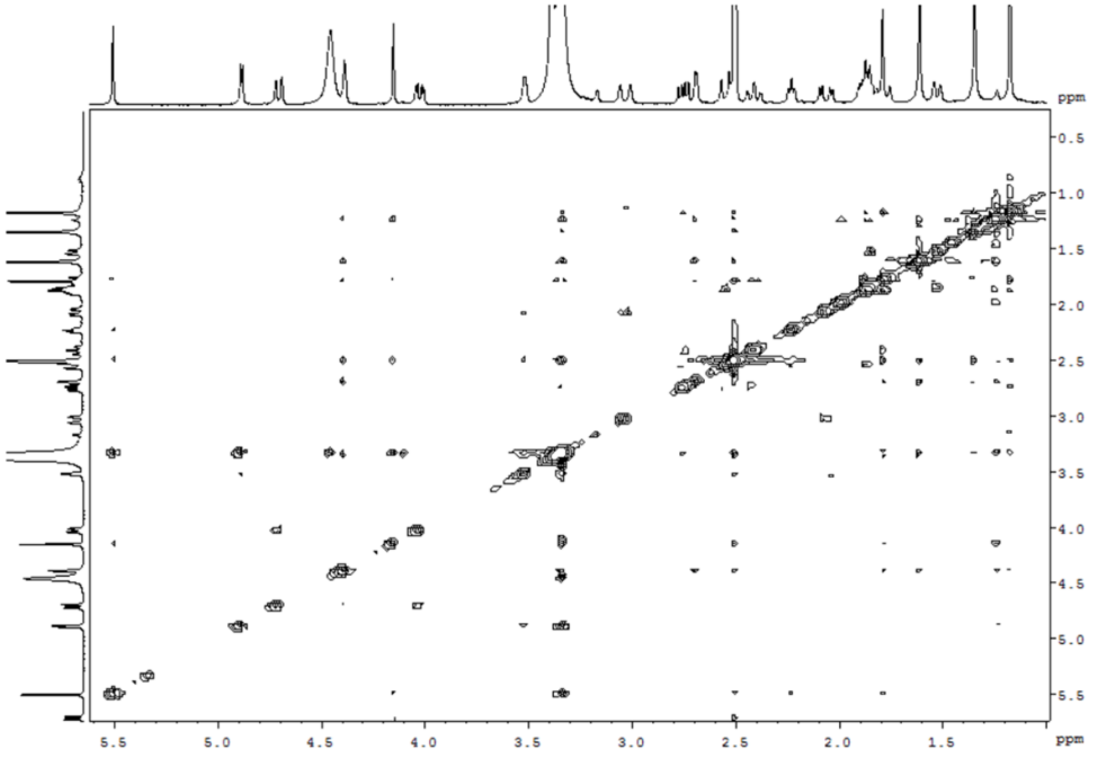

Supplementary Figure S53. Selected NOESY spectrum of **6** (600 MHz, DMSO-d_6)

Tolerance = 5.0 PPM / DBE: min = 3.0, max = 50.0
Element prediction: Off
Number of isotope peaks used for i-FIT = 3

Monoisotopic Mass, Even Electron Ions
234 formula(e) evaluated with 1 results within limits (up to 50 closest results for each mass)
Elements Used:
C: 0-38 H: 0-100 O: 0-200 Na: 0-1
SY0923pos16 24 (0.109)
1: TOF MS ES+

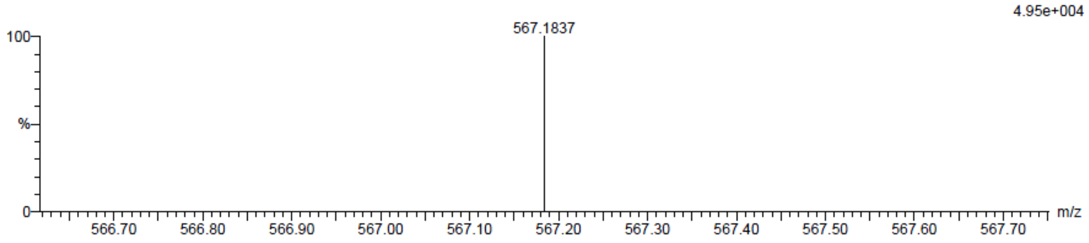

Minimum:				3.0				
Maximum:		4.0	5.0	50.0				
Mass	Calc. Mass	mDa	PPM	DBE	i-FIT	Norm	Conf(%)	Formula
567.1837	567.1842	-0.5	-0.9	12.5	34.4	n/a	n/a	C28 H32 O11 Na

Supplementary Figure S54. HRESIMS spectrum of **6**
